# Supplementary material for: RABGAP1 is a sensor that facilitates the sorting and processing of amyloid precursor protein
Source: EMBO J. 2025 Aug 26;44(19):5443–62. doi: 10.1038/s44318-025-00530-0 (PMC12489035; doi:10.1038/s44318-025-00530-0)
Supplement: Supplementary file 1 — Appendix [file 44318_2025_530_MOESM1_ESM.pdf]

Appendix for RABGAP1 acts as a sensor to facilitate sorting and processing of amyloid precursor protein

Contents

Appendix Figure S1 1
Appendix Figure S2 2
Appendix Figure S3 3
Appendix Figure S4 4
Appendix Figure S5 5
Appendix Figure S6 6
Appendix Figure S7 7
Appendix Figure S8 8
Appendix Figure S9 9
Appendix Figure S10 10

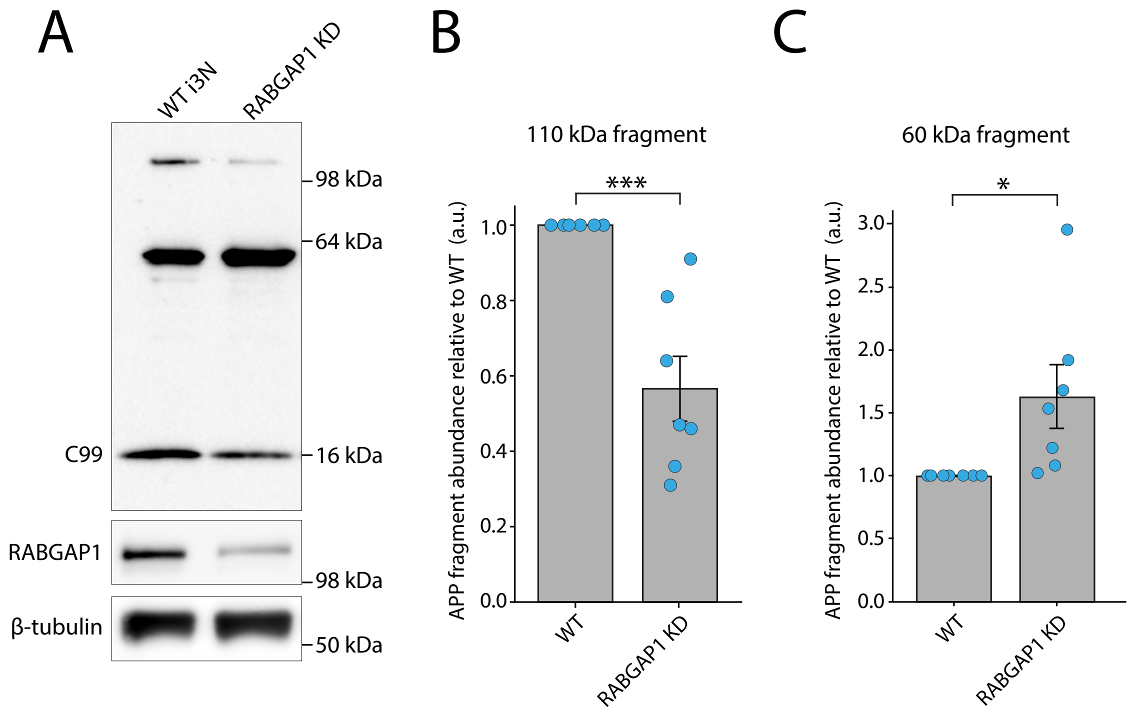

Appendix Figure S1. APP processing is significantly altered in RABGAP1 KD i3 neurons.
Immunoblotting of endogenous APP processing in RABGAP1 KD i3 neurons. Neurons were lysed at day 15 of differentiation. (**A**) Full immunoblot, as shown in Figure 5E. APP processing levels were assessed using the D54D2 antibody (Cell Signalling Technologies, 8243). β-tubulin was used as a loading control. RABGAP1 was probed to monitor RABGAP1 KD efficiency. (**B**) Quantification of 110 kDa peptide, shown in (A). (**C**) Quantification of 60 kDa peptide, shown in (A). N=7 biological repeats. Bars represent the mean ± SEM (B-C). Statistical significance was assessed using an unpaired t-test. ***$p \leq 0.001$; *$p \leq 0.05$.

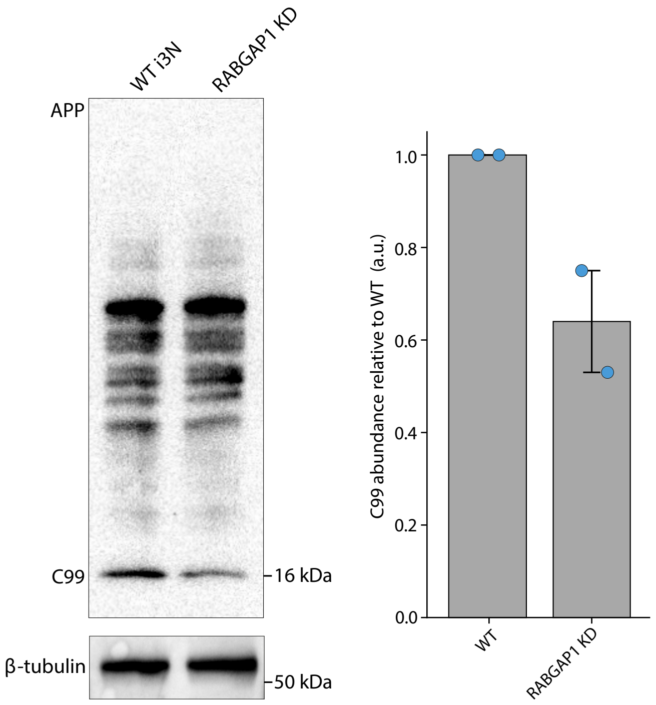

Appendix Figure S2. C99 levels detected by 82E1 antibody.
Immunoblotting of endogenous APP processing in RABGAP1 KD i3 neurons using an alternative C99 antibody, 82E1 (Stratech Scientific, 10323). Neurons were lysed at day 15 of differentiation.

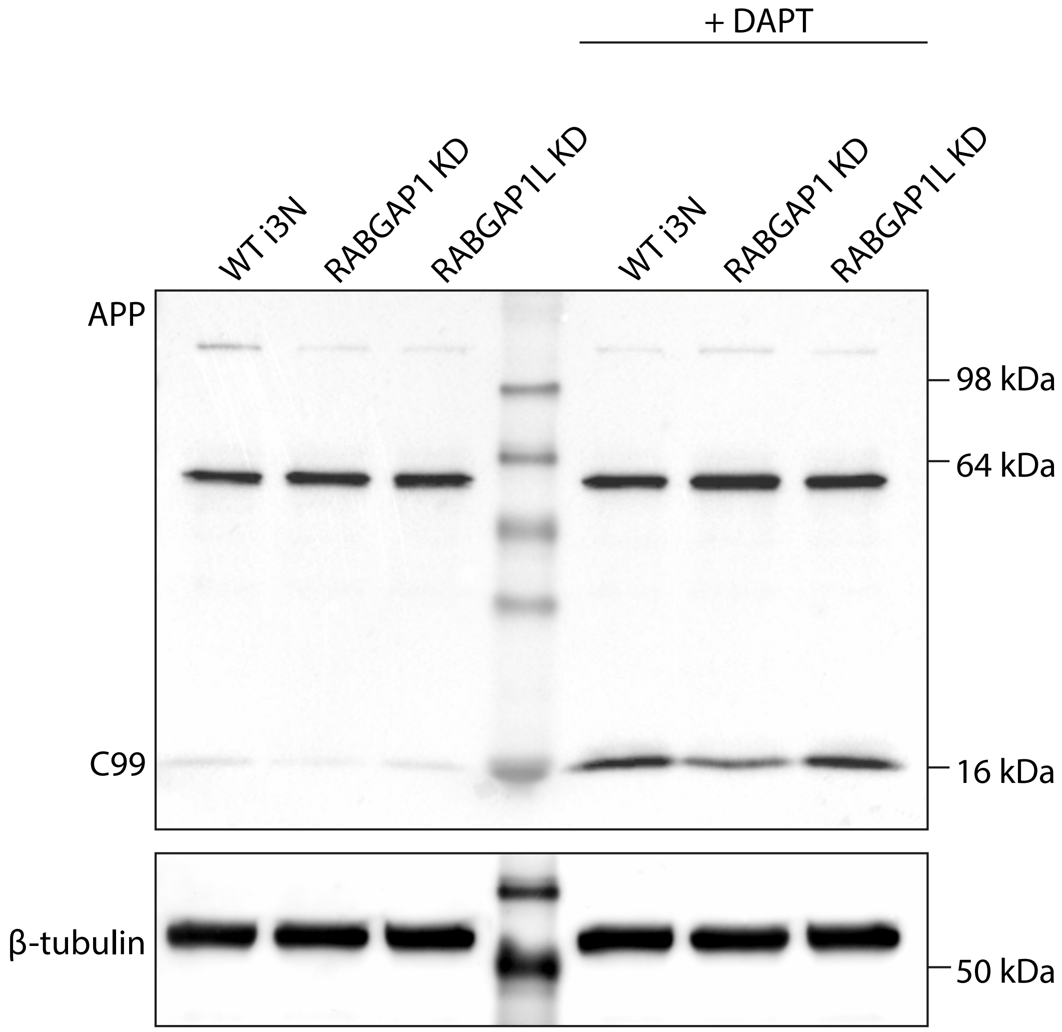

Appendix Figure S3. DAPT treatment caused accumulation of the 16 kDa peptide.
Immunoblotting of endogenous APP processing in WT, RABGAP1 KD, and RABGAP1L KD i3 neurons in the presence of DAPT, a γ-secretase inhibitor, using the D54D2 antibody (Cell Signalling Technologies, 8243). Cells were treated with 25 µM of DAPT for 24 hours before lysis.

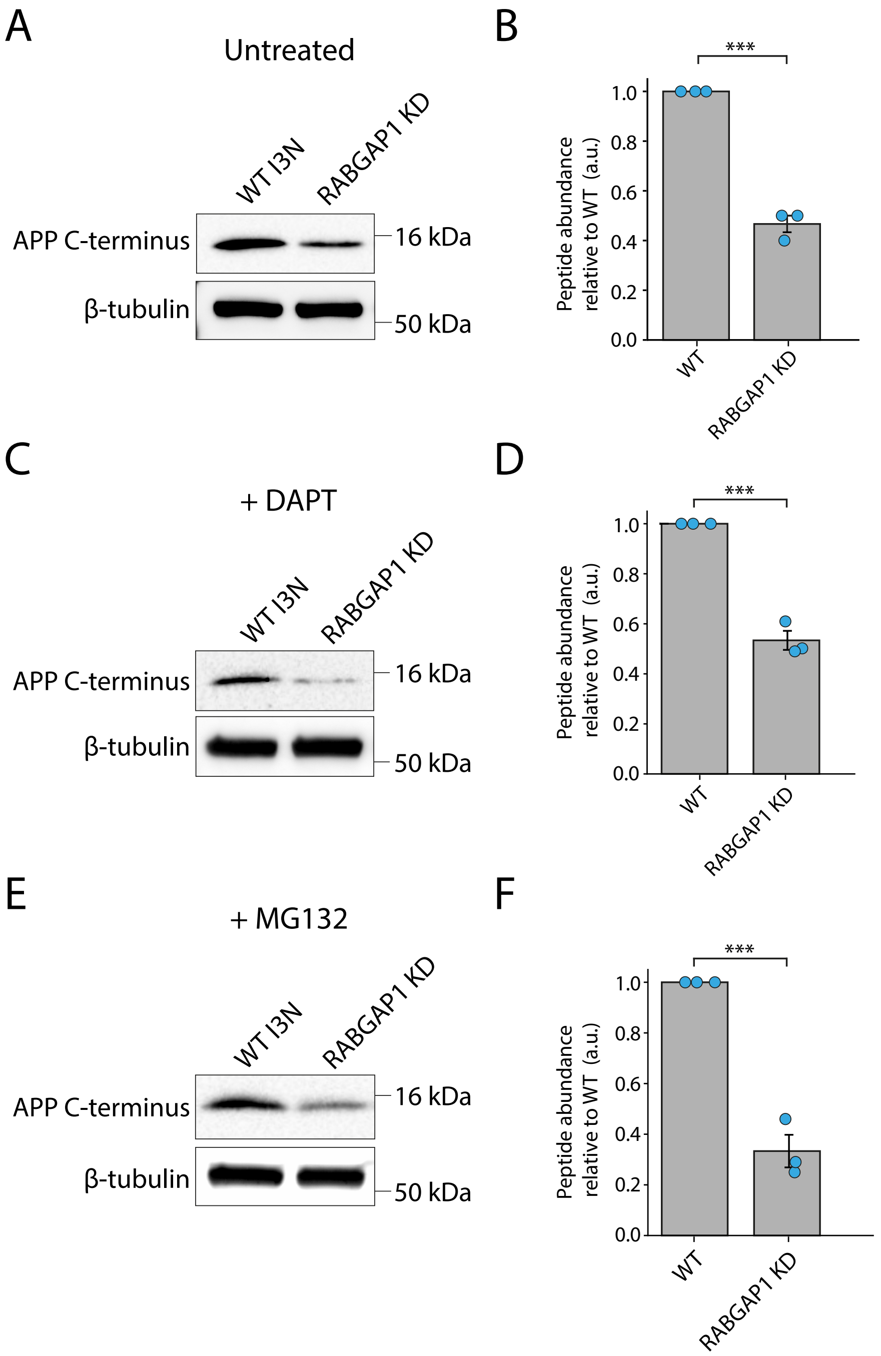

Appendix Figure S4. APP processing defects in RABGAP1 KD i3 neurons are also detected by a C-terminal AICD antibody.

APP processing defects observed upon RABGAP1 KD were also detected using the C-terminal β-amyloid CT695 antibody (Invitrogen, 51-2700) that binds to the AICD fragment of APP. (**A**) Immunoblotting of APP processing in WT and RABGAP1 KD i3 neurons. Neurons were lysed at day 15 of differentiation. β-tubulin was used as a loading control. (**B**) Quantification of the 16 kDa APP processing fragment, shown in (A). (**C**) Immunoblotting of WT and RABGAP1 KD i3 neurons in the presence of DAPT, a γ-secretase inhibitor. Cells were treated with 25 µM of DAPT for 24 hours before lysis. (**D**) Quantification of the 16 kDa APP processing fragment, shown in (C). (**E**) Immunoblotting of WT and RABGAP1 KD i3 neurons in the presence of proteasome inhibitor, MG132. Cells were treated with 10 µM MG132 for 1 hour before lysis. (**F**) Quantification of the 16 kDa APP processing fragment, shown in (E). N=3 biological repeats. Bars represent the mean ± SEM (B-F). Statistical significance was assessed using an unpaired t-test. ***p $\leq$0.001.

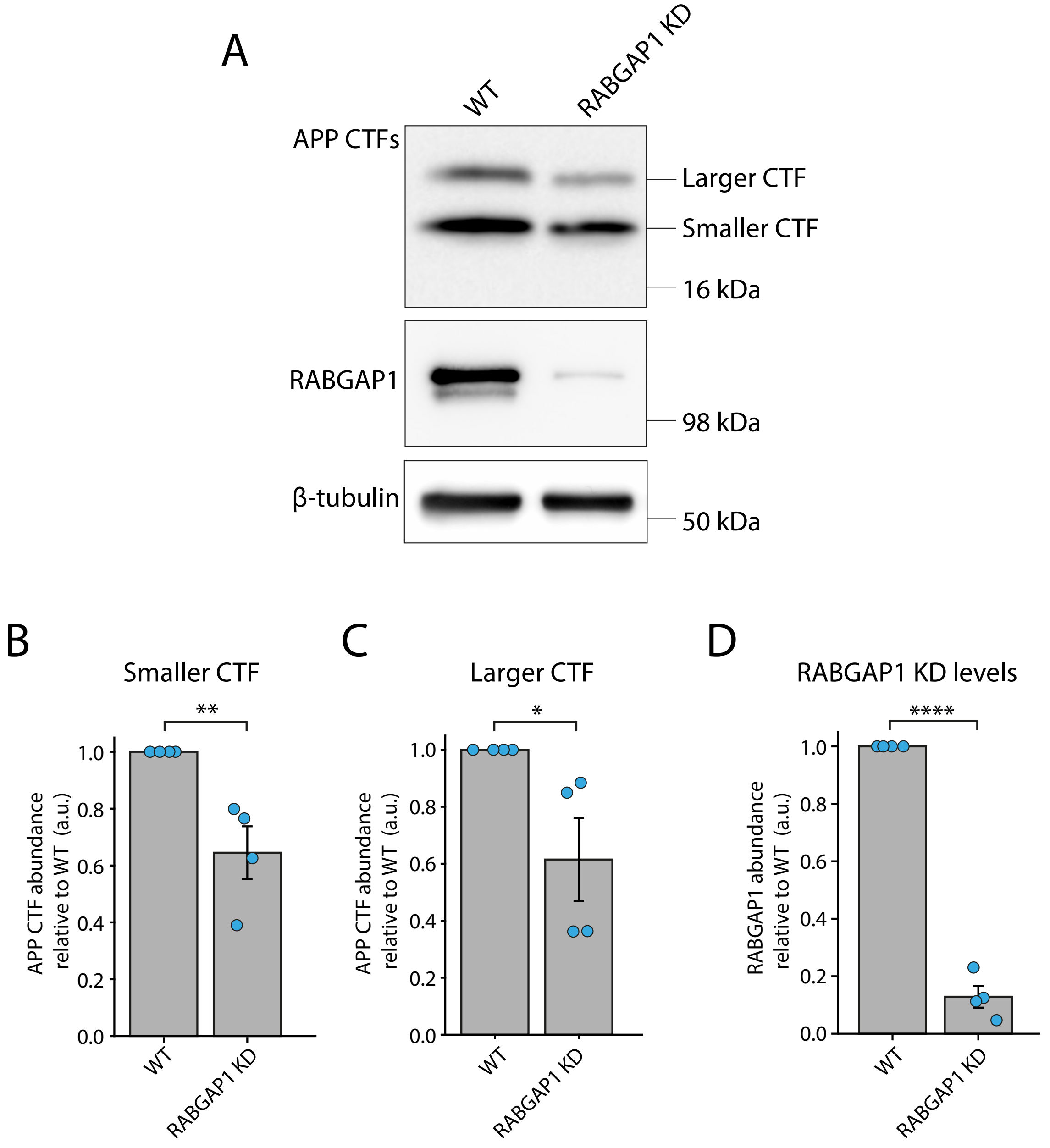

Appendix Figure S5. APP CTF abundance is reduced in primary neurons upon RABGAP1 KD.

(**A**) shRNA-mediated KD of RABGAP1 in DIV21 primary rat hippocampal neurons. Immunoblotting was used to assess APP processing defects in RABGAP1 KD neurons compared to WT, using the APP AICD antibody (Invitrogen, 51-2700). β-tubulin was used as a loading control. (**B**) Quantification of the lower molecular weight CTF (smaller CTF), as seen in (A). (**C**) Quantification of the higher molecular weight CTF (larger CTF), as seen in (A). (**D**) Quantification of RABGAP1 KD efficiency using shRNA. N=4 biological repeats. Bars represent the mean ± SEM (B-D). Statistical significance was assessed using an unpaired t-test. ****p $\leq$0.0001; **p $\leq$0.01; *p $\leq$0.05.

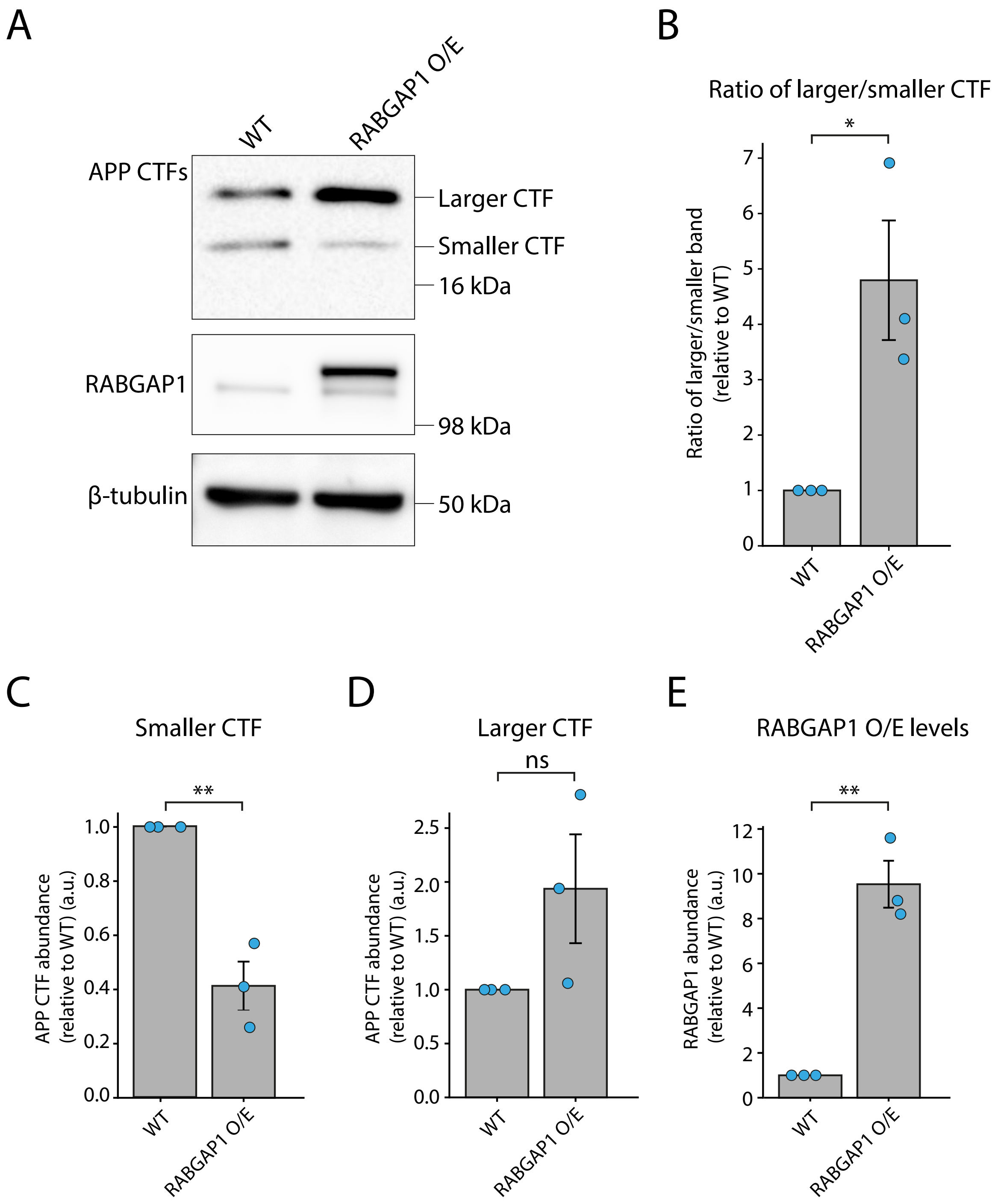

Appendix Figure S6. APP CTF abundance is affected in primary neurons upon RABGAP1 overexpression.
(**A**) RABGAP1 overexpression in DIV21 primary rat hippocampal neurons. RABGAP1 overexpression was achieved through the addition of 100 µl/well of 20x RABGAP1-mEmerald lentivirus on DIV5. Immunoblotting was used to assess APP processing defects in RABGAP1 KD neurons compared to WT, using the C-terminal APP antibody (Invitrogen, 51-2700). β-tubulin was used as a loading control. (**B**) Quantification of the ratio of two CTFs in RABGAP1 KD neurons, relative to WT, as seen in (A). (**C**) Quantification of the lower molecular weight CTF, relative to WT neurons, as shown in (A). (**D**) Quantification of the higher molecular weight CTF, relative to WT, as seen in (A). (**E**) Quantification of RABGAP1 overexpression relative to WT cells. N=4 biological repeats. Bars represent the mean ± SEM (B-E). Statistical significance was assessed using an unpaired t-test. **$p \leq 0.01$; *$p \leq 0.05$. ns = not significant.

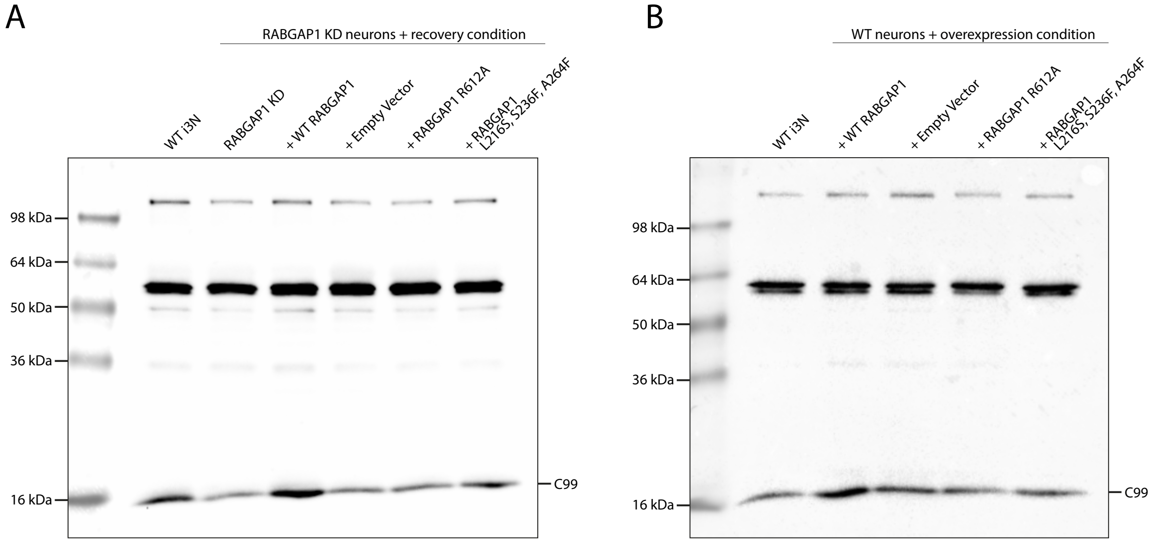

Appendix Figure S7. Full blots of complementation and overexpression assays shown in Figure 6.
(**A**) The full immunoblot of Figure 6A where RABGAP1 KD neurons were complemented with either WT RABGAP1, a GAP-deficient mutant (R612A) or a mutant that no longer interacts with the APP tail (L216S, S236F, A264F). (**B**) The full immunoblot of RABGAP1 overexpression in i3 neurons, as shown in Figure 6C. Stable iPSC lines were generated with overexpression of either WT RABGAP1, a RABGAP1 GAP-deficient mutant (R612A) or a RABGAP1 mutant that is predicted to no longer interact with the APP tail (L216S, S236F, A264F). The cell lines were differentiated and endogenous C99 levels were assessed using the D54D2 antibody (Cell Signalling, 8243).

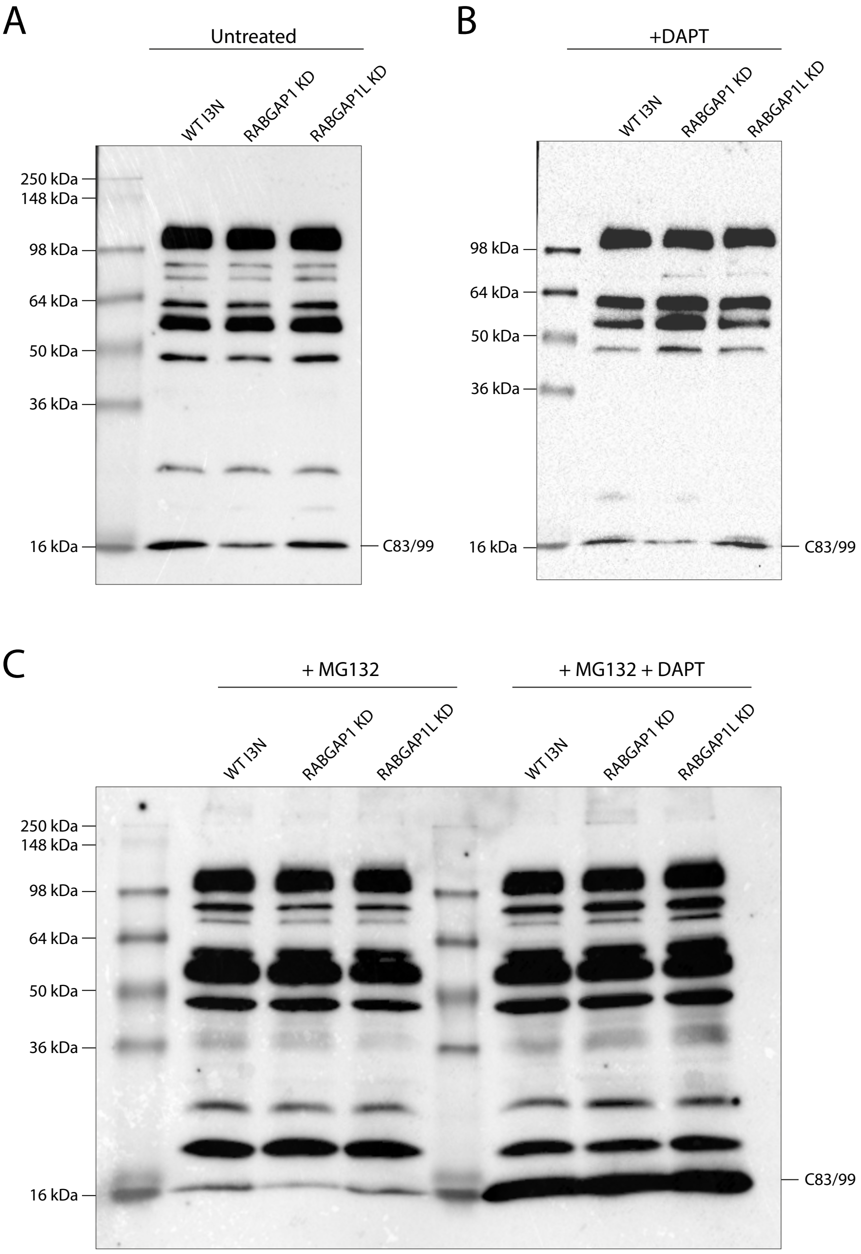

Appendix Figure S8. Full blots of APP processing defects detected in RABGAP1 KD i3 neurons by a C-terminal AICD antibody.

Full immunoblots of endogenous APP processing defects observed upon RABGAP1 KD in i3 neurons, using the C-terminal β-amyloid CT695 antibody (Invitrogen, 51-2700), as shown in Figure S4. (**A**) Immunoblotting of endogenous APP processing in WT and RABGAP1 KD i3 neurons in untreated neurons. (**B**) Immunoblotting of endogenous APP processing in WT and RABGAP1 KD i3 neurons treated with a γ-secretase inhibitor, DAPT. Cells were treated with 25 µM of DAPT for 24 hours before lysis. (**C**) Immunoblotting of endogenous APP processing in WT and RABGAP1 KD i3 neurons, treated with either MG132, a proteasome inhibitor, only or both MG132 and DAPT in combination. Cells were treated with 25 µM of DAPT for 24 hours and 10 µM MG132 for 1 hour before lysis.

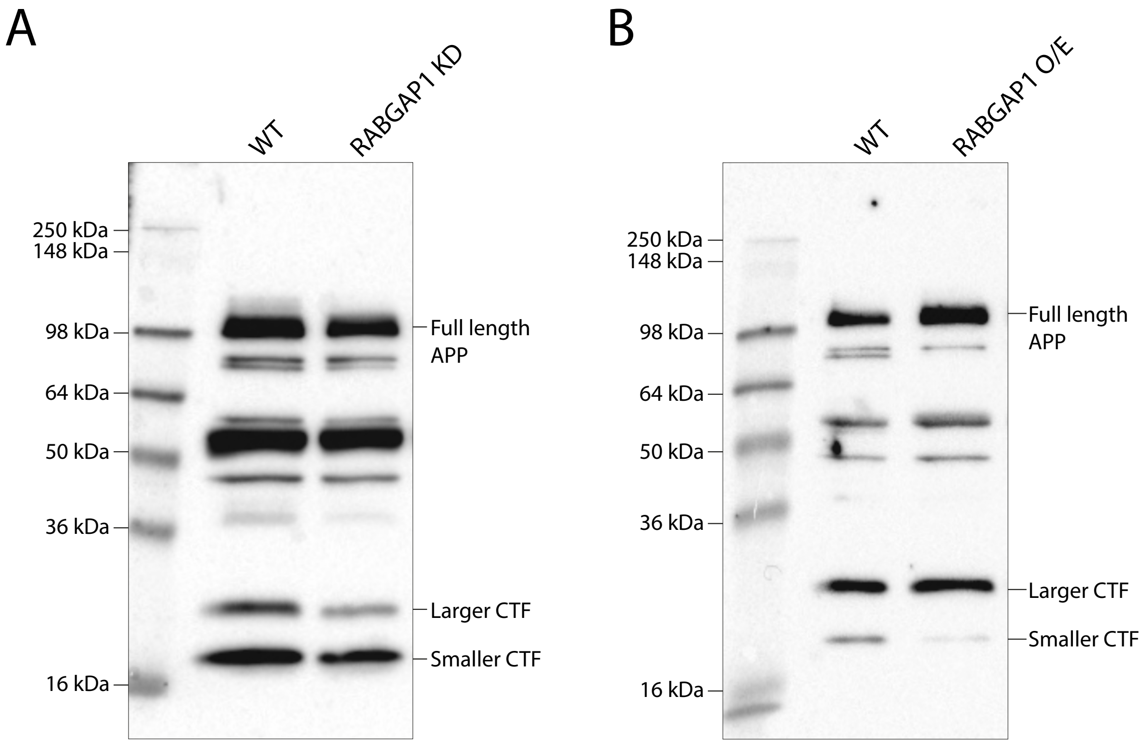

Appendix Figure S9. Full blots of APP processing defects detected in RABGAP1 KD primary rat hippocampal neurons. Full immunoblots of APP processing defects observed upon either RABGAP1 KD (**A**) or RABGAP1 overexpression (**B**), as shown in Figures S5 and S6. Endogenous APP is detected using the C-terminal β-amyloid CT695 antibody (Invitrogen, 51-2700) that binds to the AICD fragment of APP.

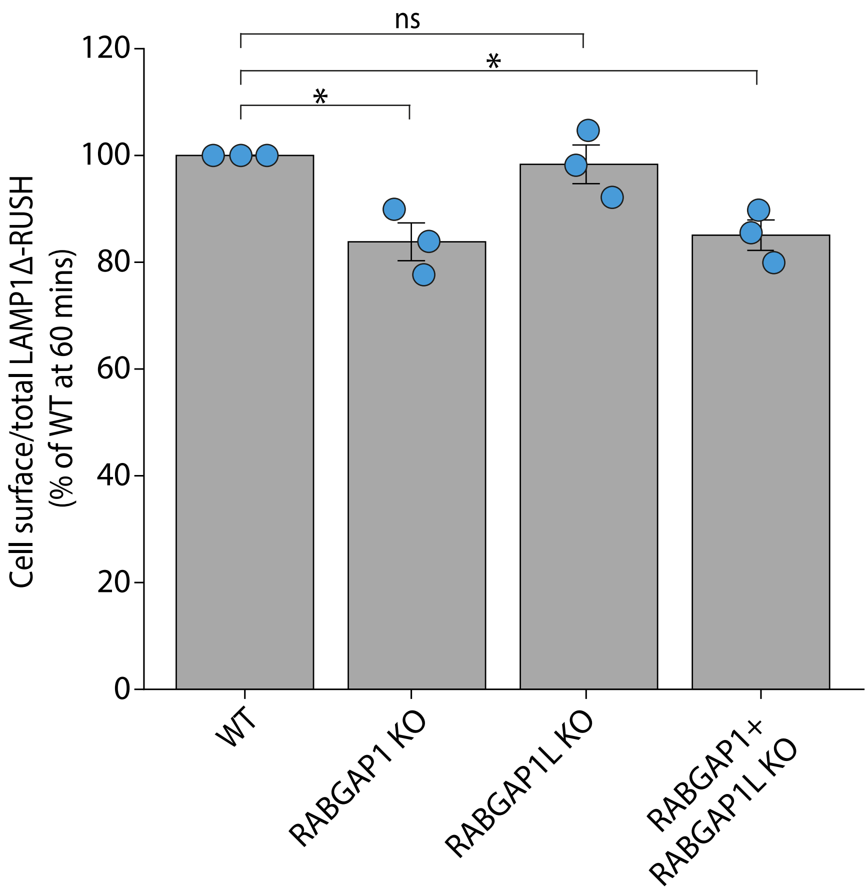

Appendix Figure S10. RABGAP1 KO has only a minor effect on global protein trafficking through the secretory pathway. Kinetic trafficking assay tracking the synchronised transport of a model secretory cargo, LAMP1Δ-RUSH, which moves from the ER to the Golgi and ultimately accumulates at the plasma membrane. The y-axis represents the percentage of cargo detected at the cell surface relative to total cellular expression, measured 60 minutes after ER release. N=3 biological repeats. Bars represent the mean ± SEM. Statistical significance was assessed using one-way ANOVA followed by Tukey's Honest Significant Difference (HSD) multiple comparisons post-hoc test (FWER = 0.05). *$p \leq 0.05$; ns = not significant.
