# Supplementary figures and images for: RABGAP1 is a sensor that facilitates the sorting and processing of amyloid precursor protein
Source: EMBO J. 2025 Aug 26;44(19):5443–62. doi: 10.1038/s44318-025-00530-0 (PMC12489035; doi:10.1038/s44318-025-00530-0)

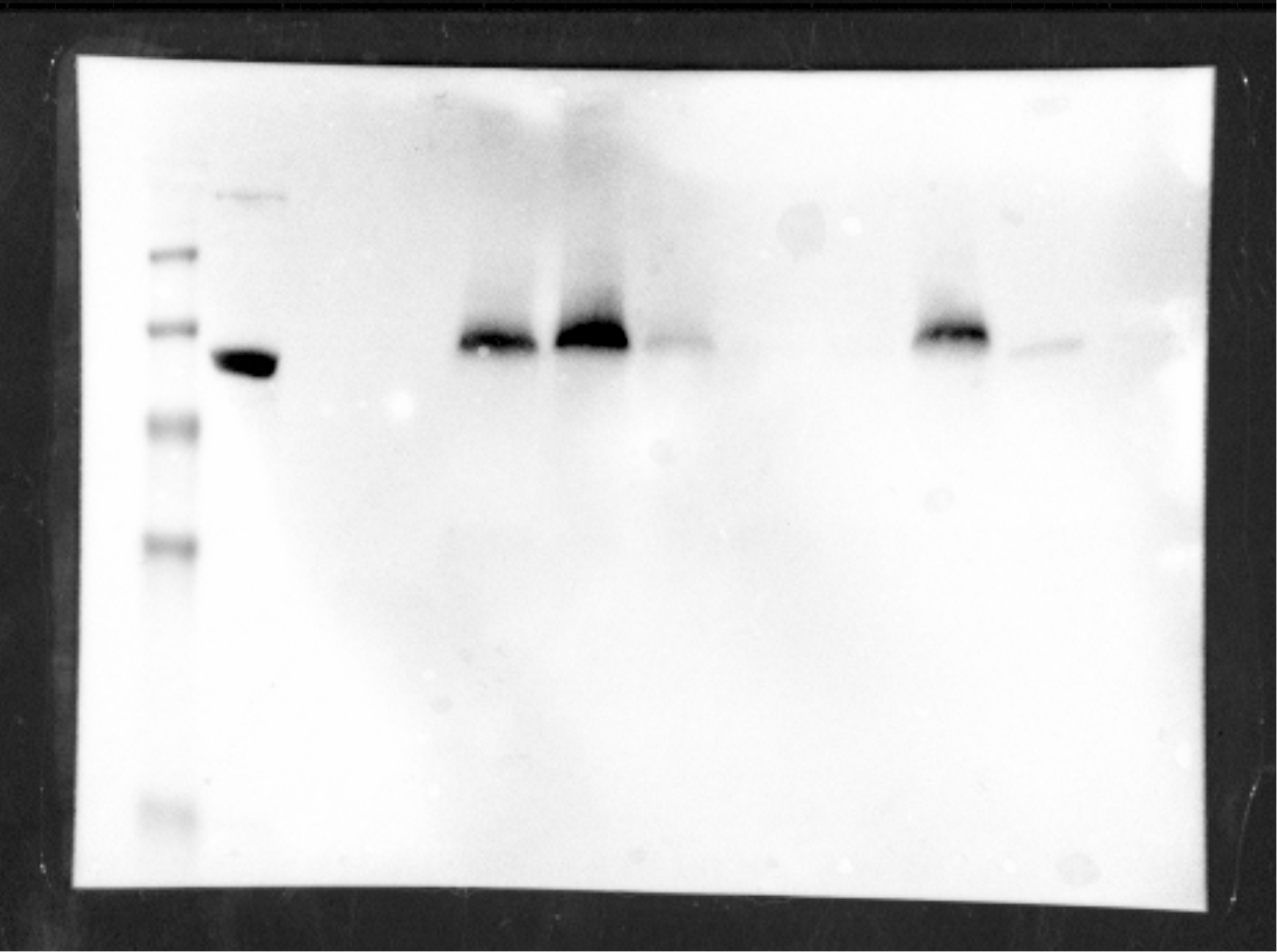

Supplement: Supplementary file 8 — Source data Fig. 2 [file 44318_2025_530_MOESM8_ESM.zip › Figure 2/2B/SNX17-blot.tif]

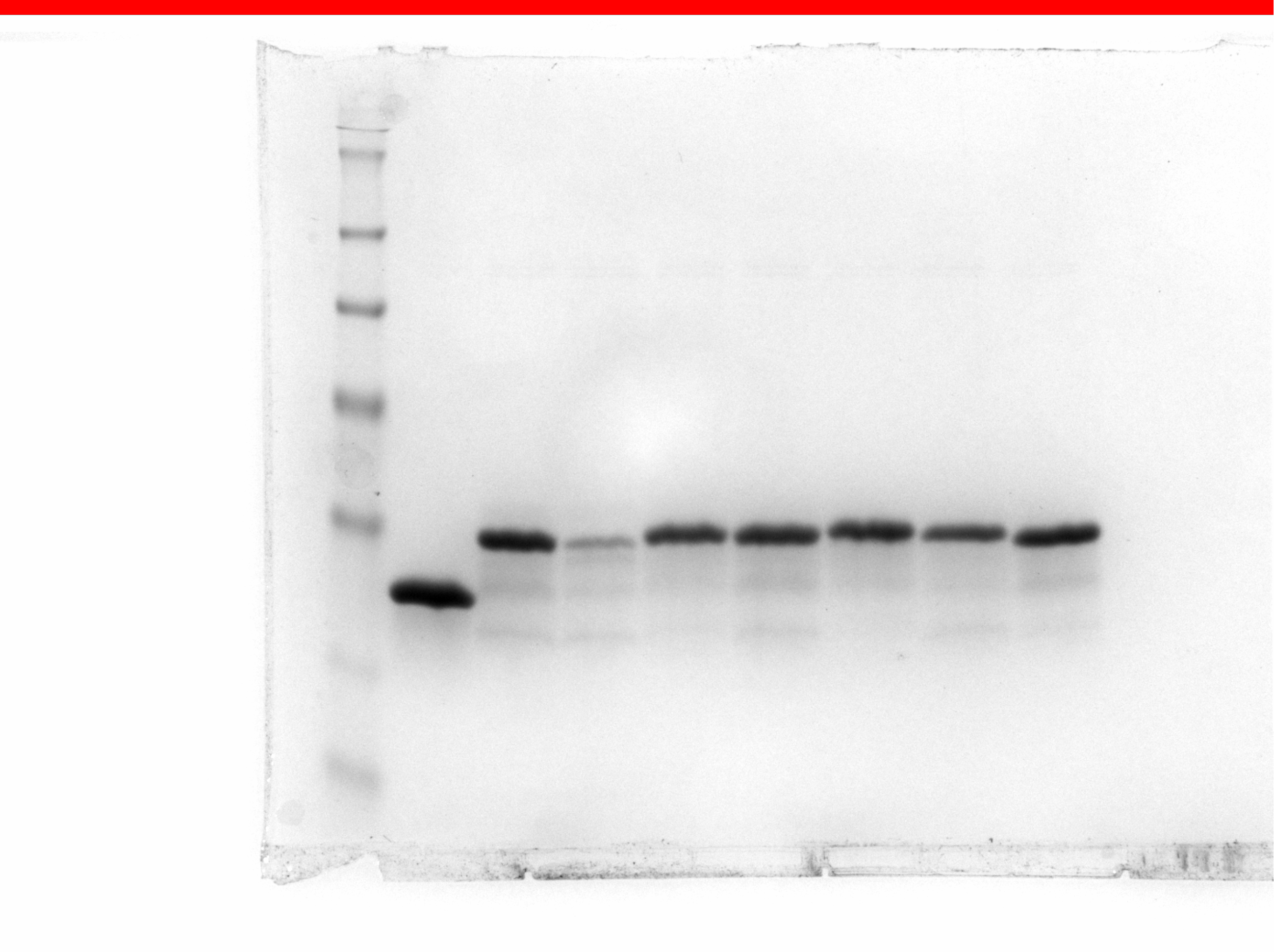

Supplement: Supplementary file 8 — Source data Fig. 2 [file 44318_2025_530_MOESM8_ESM.zip › Figure 2/2B/Coomassie.tif]

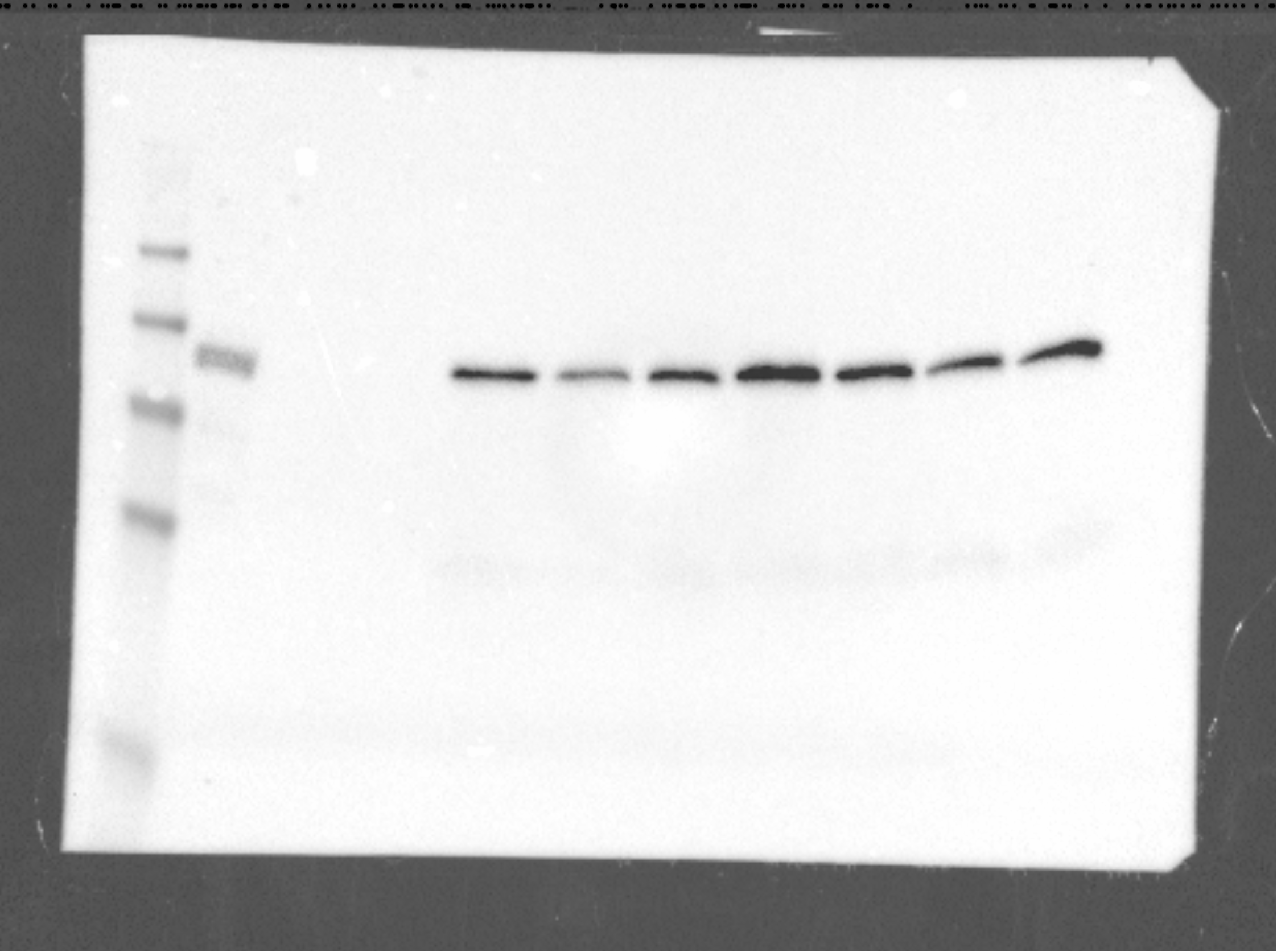

Supplement: Supplementary file 8 — Source data Fig. 2 [file 44318_2025_530_MOESM8_ESM.zip › Figure 2/2B/PDLIM7-blot.tif]

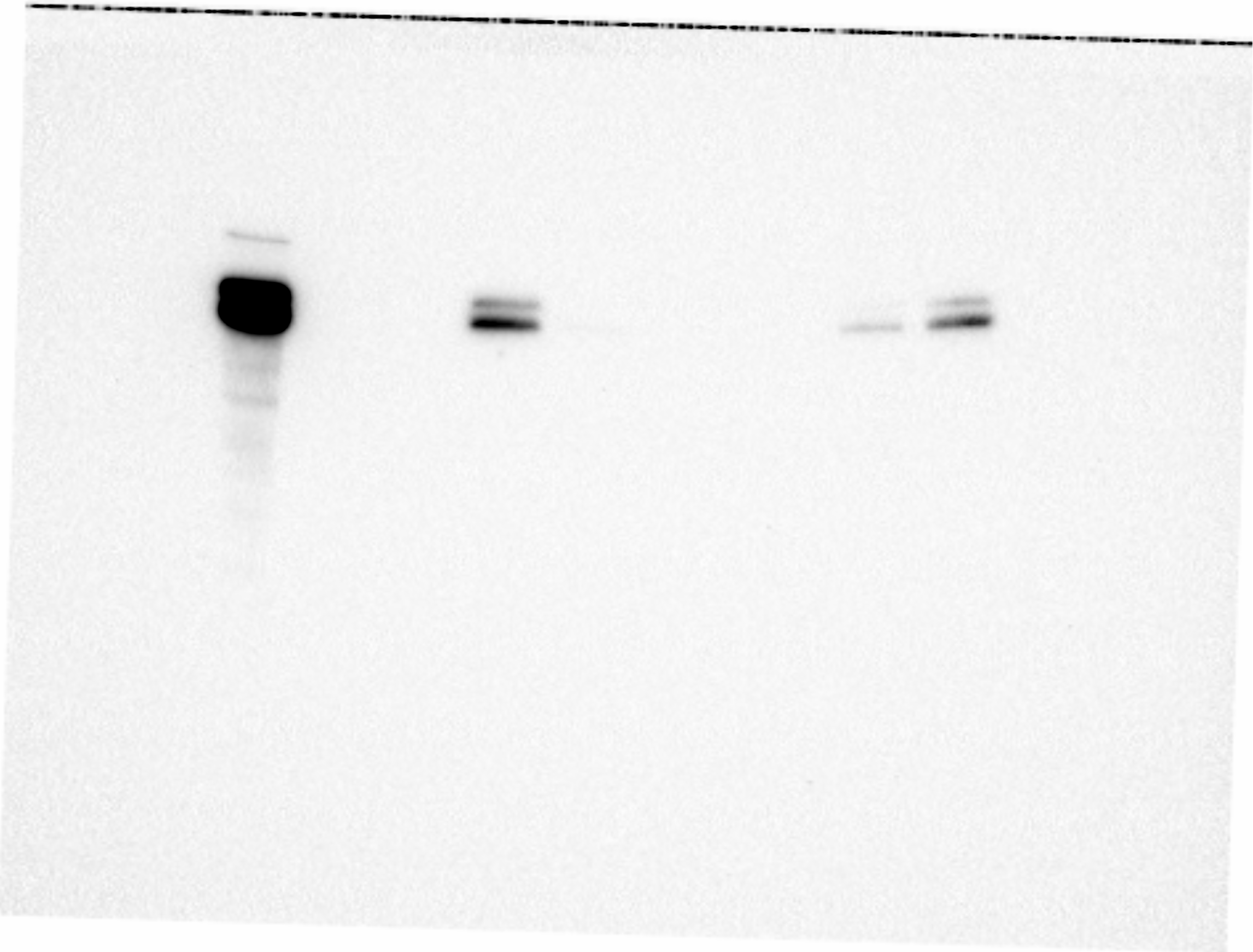

Supplement: Supplementary file 8 — Source data Fig. 2 [file 44318_2025_530_MOESM8_ESM.zip › Figure 2/2B/NUMB-blot.tif]

Coomassie

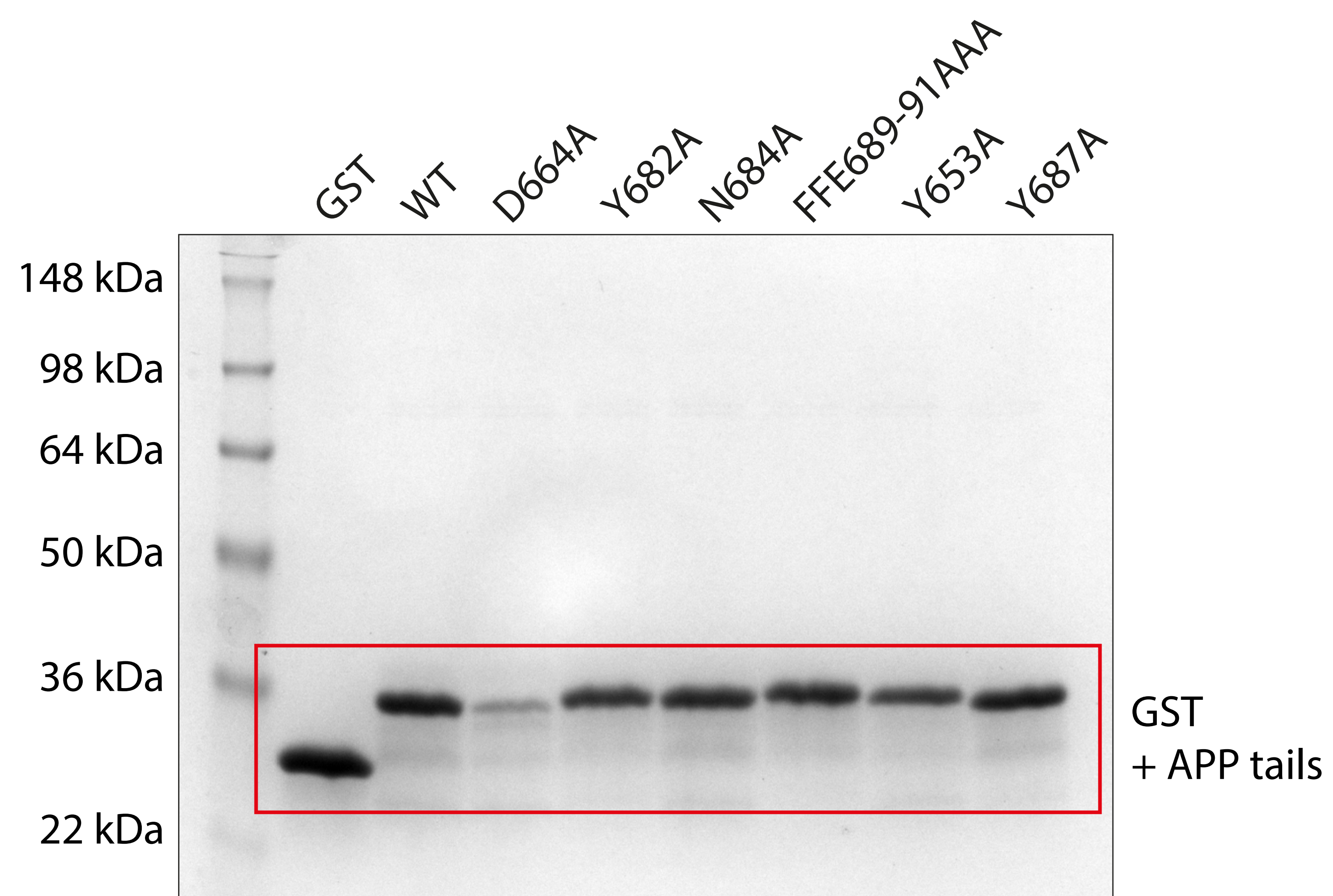

RABGAP1 blot

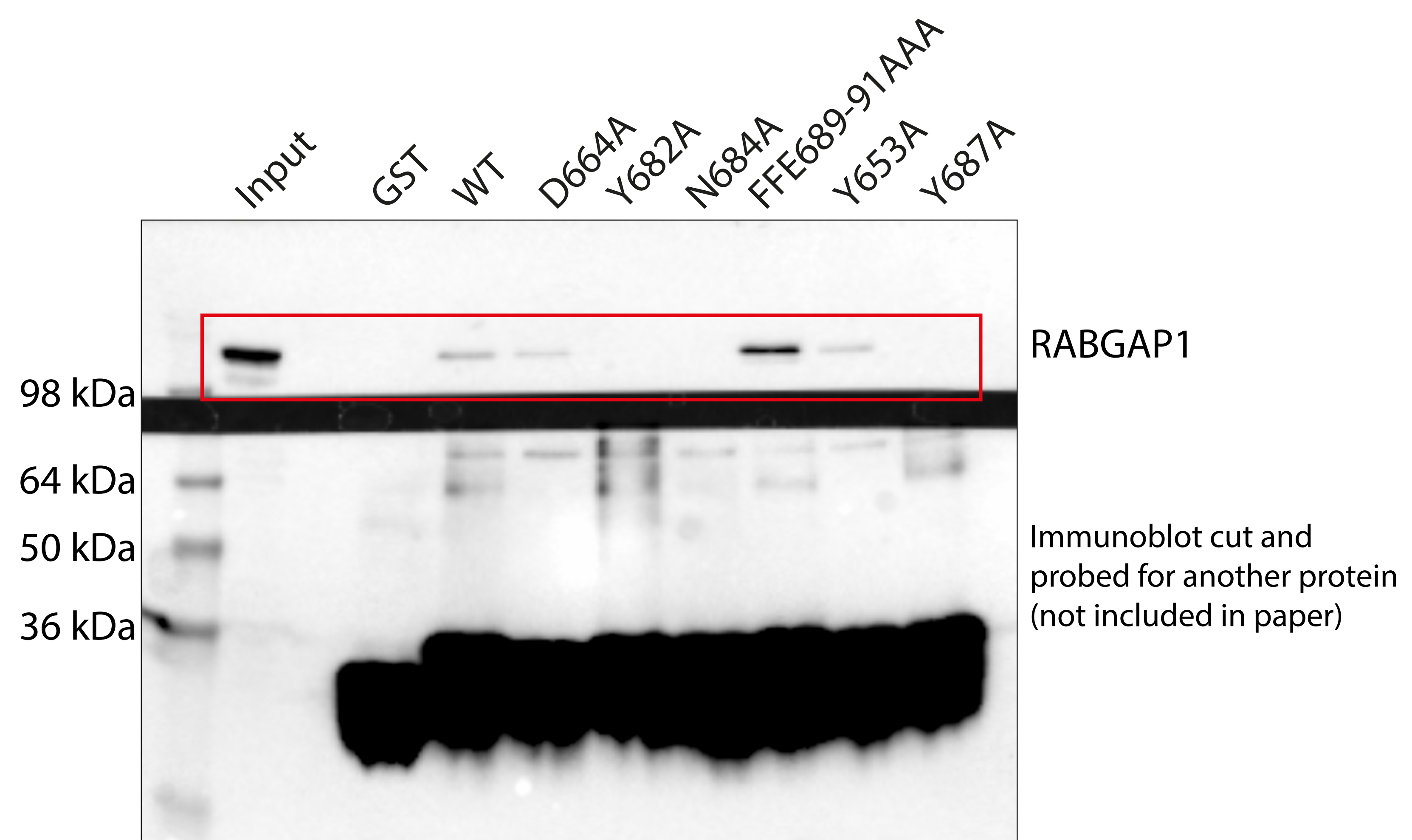

SNX17 blot

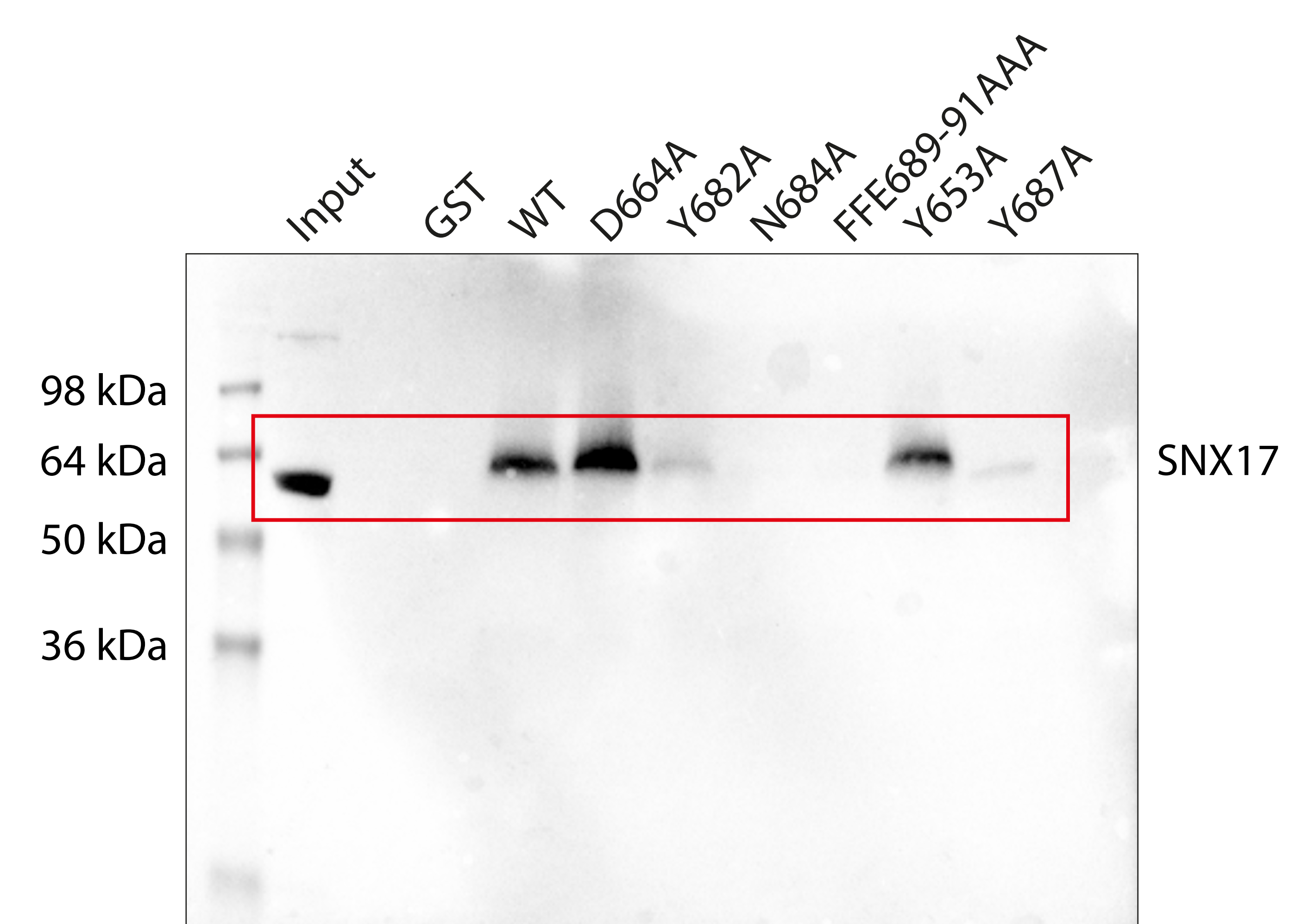

PDLIM7 blot

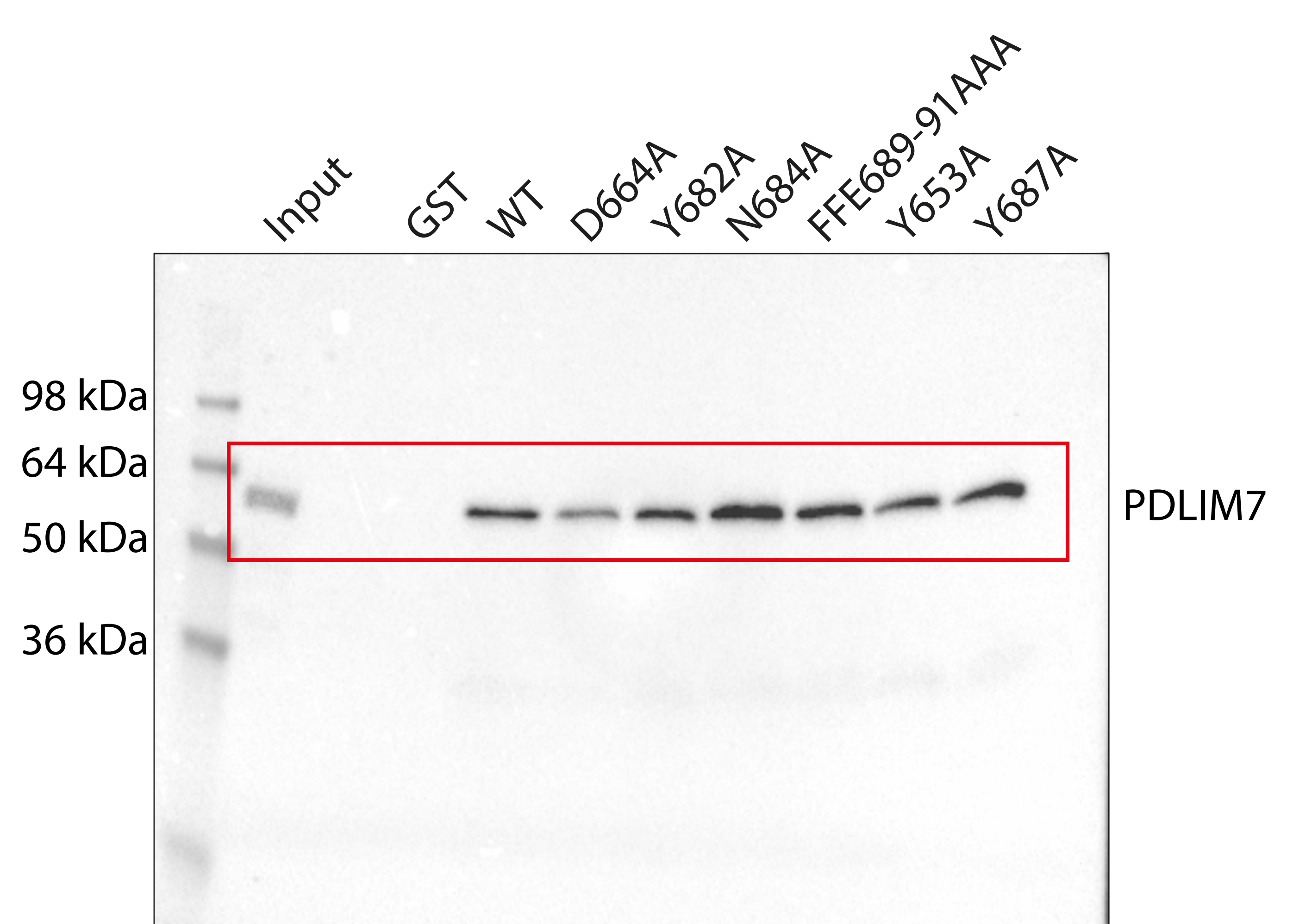

NUMB blot

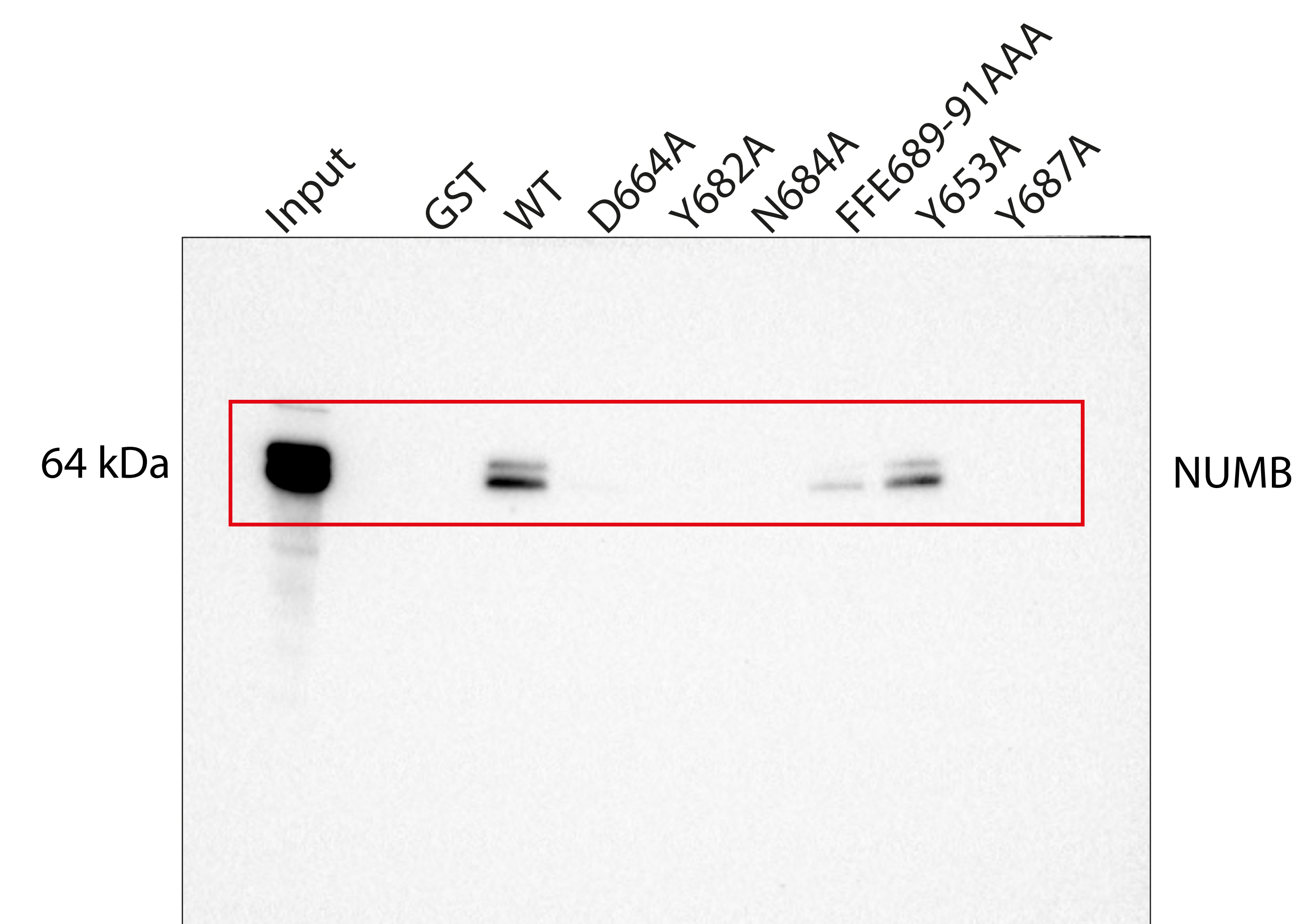

Supplement: Supplementary file 8 — Source data Fig. 2 [file 44318_2025_530_MOESM8_ESM.zip › Figure 2/2B/Figure 2B-blots.pdf]

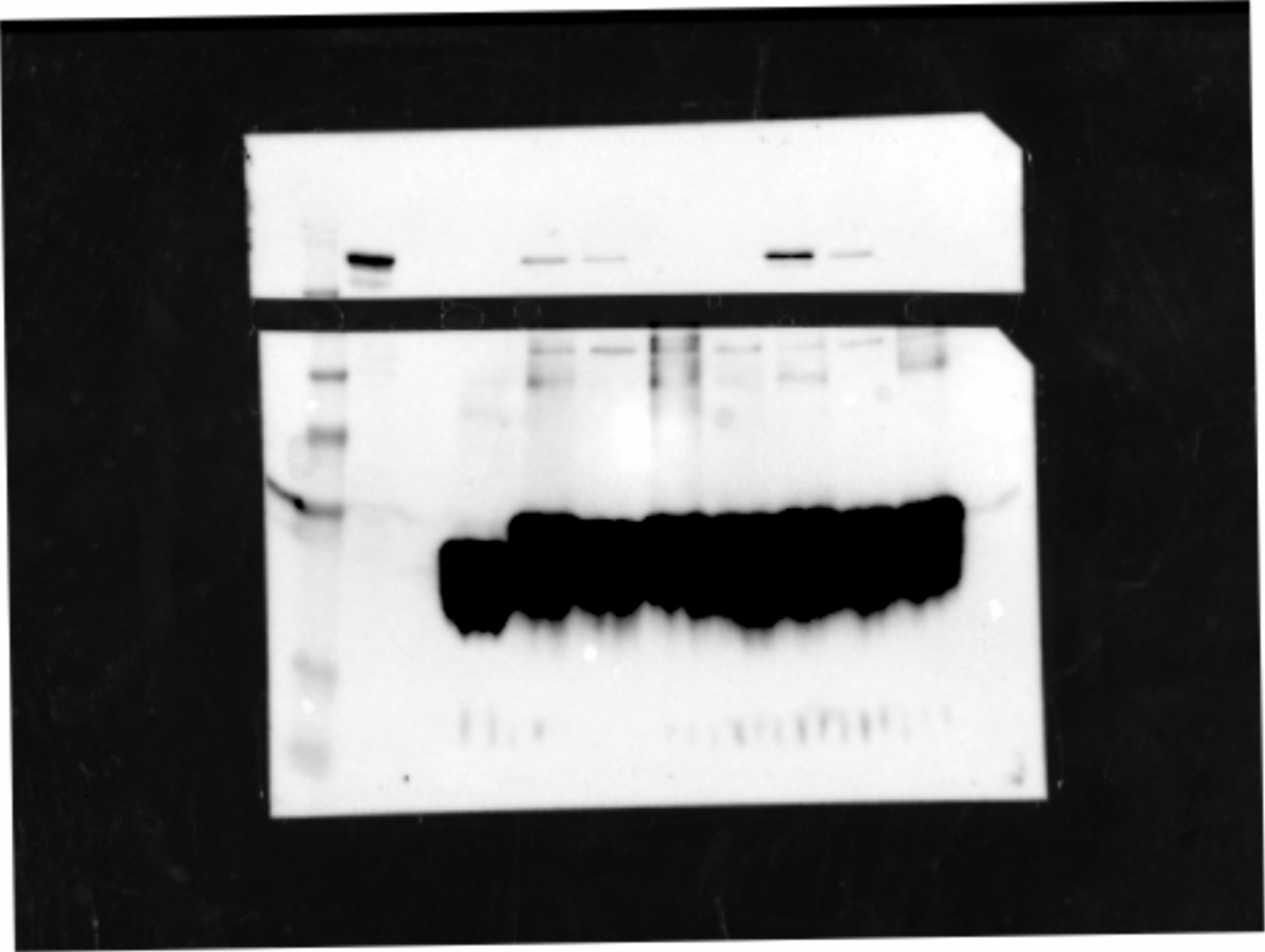

Supplement: Supplementary file 8 — Source data Fig. 2 [file 44318_2025_530_MOESM8_ESM.zip › Figure 2/2B/RABGAP1-blot.tif]

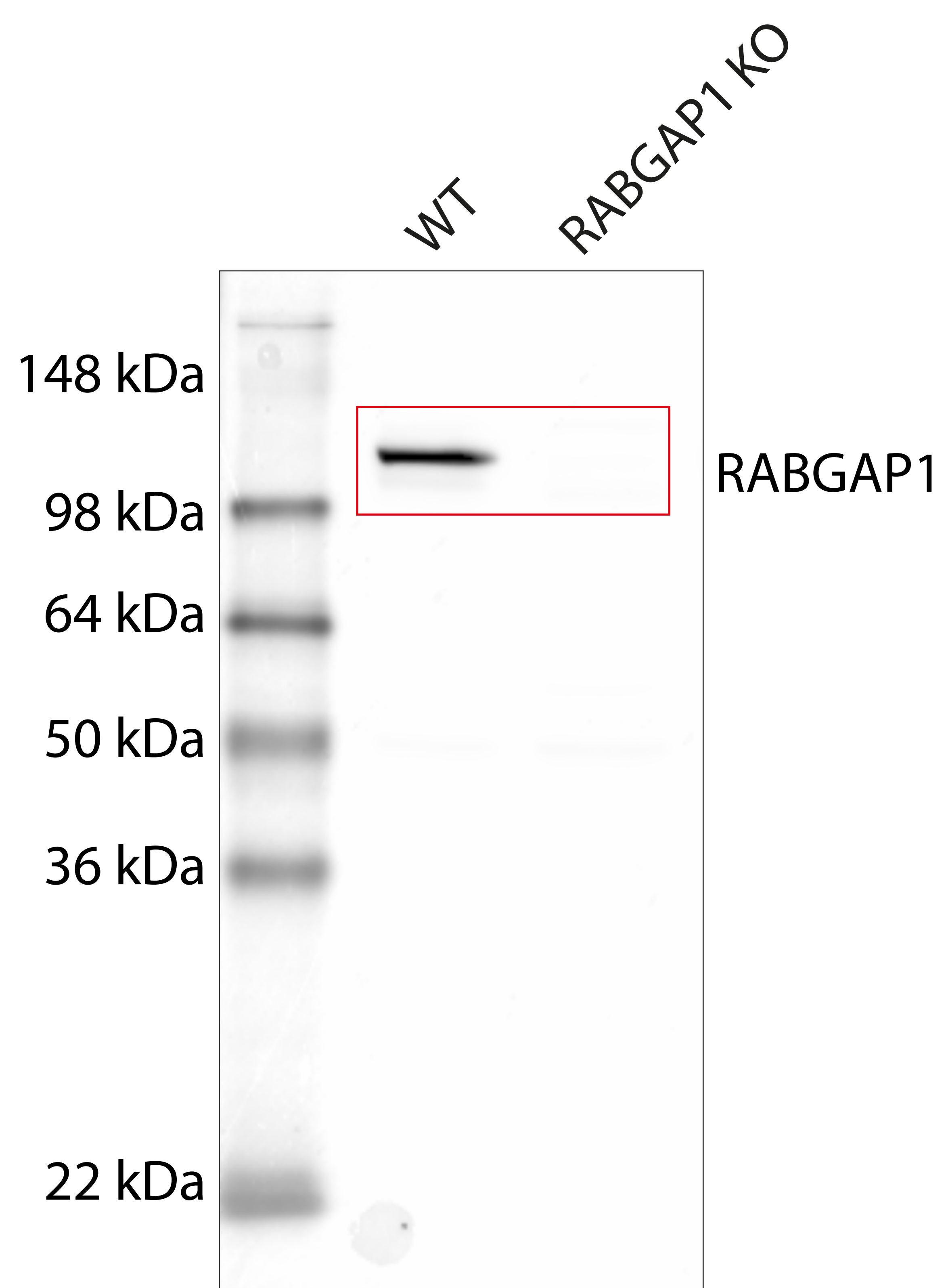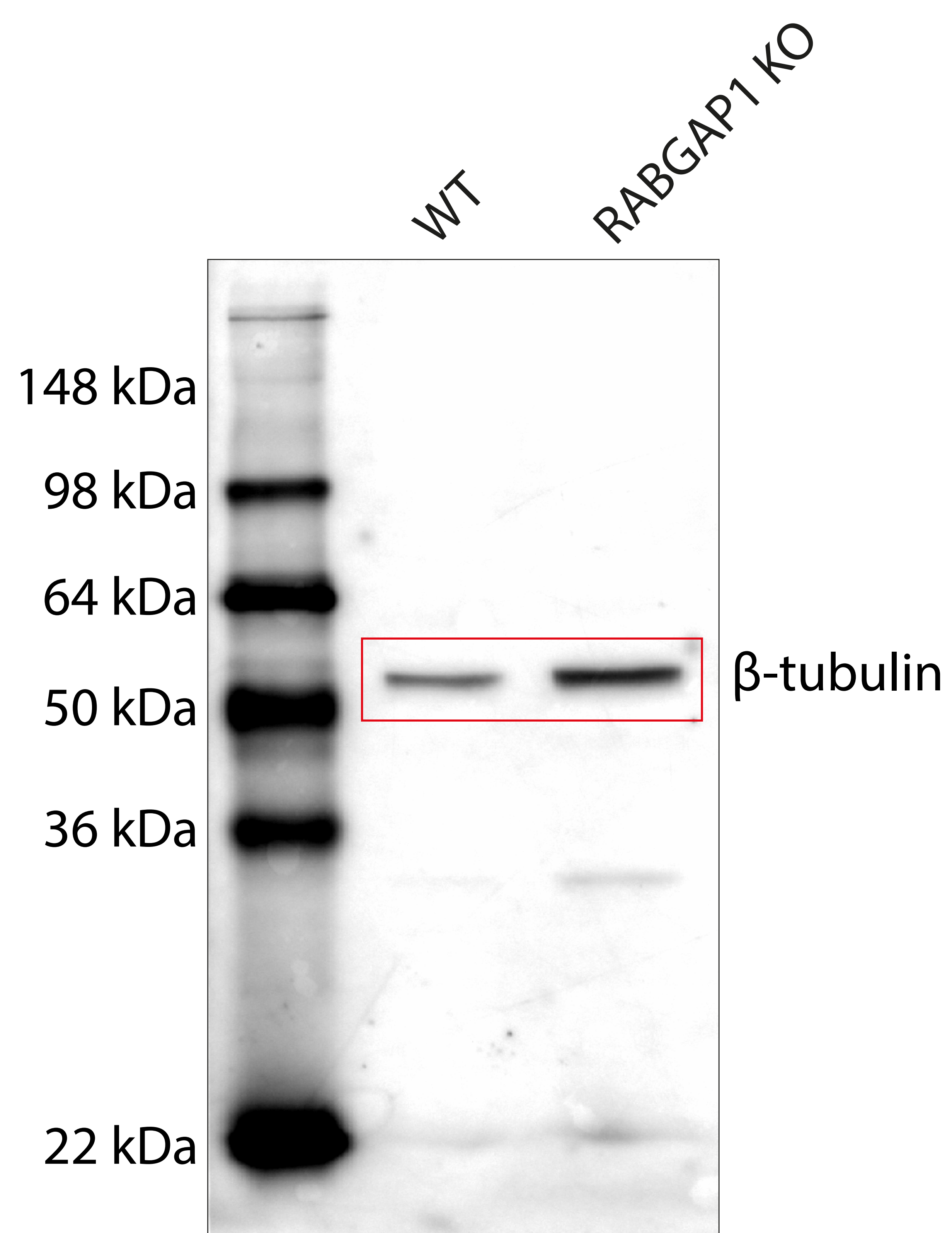

Supplement: Supplementary file 10 — Source data Fig. 4 [file 44318_2025_530_MOESM10_ESM.zip › Figure 4/4B/RABGAP1-KO-validation-blot.pdf]

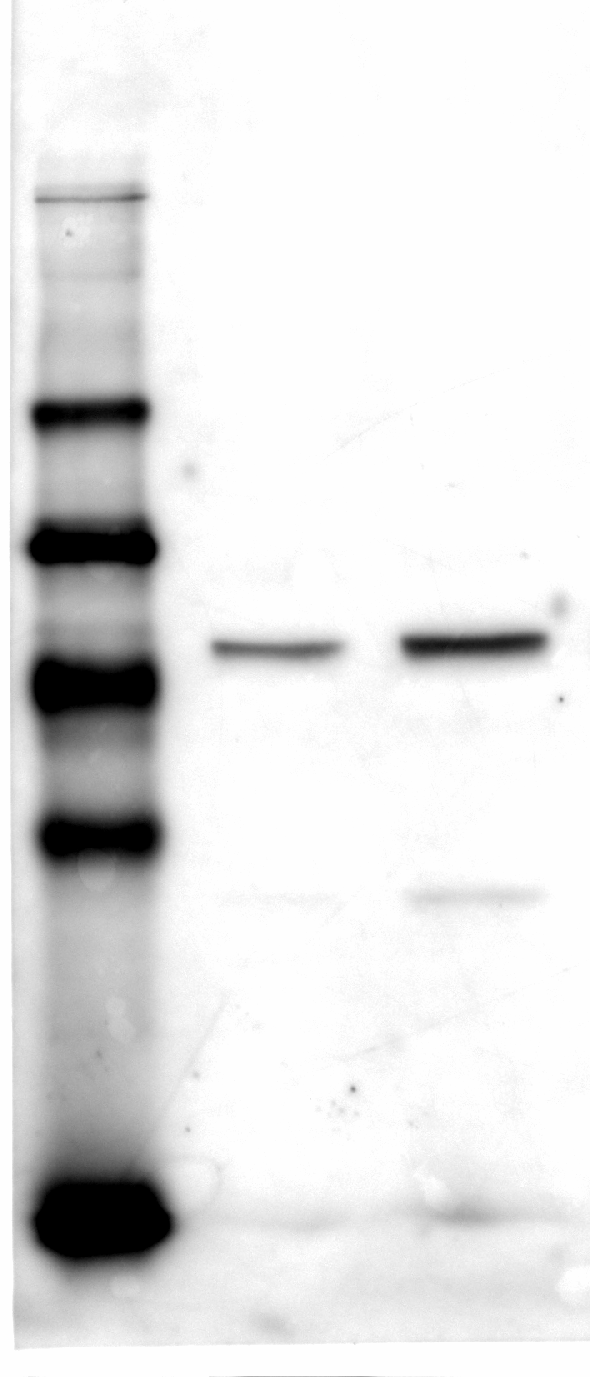

Supplement: Supplementary file 10 — Source data Fig. 4 [file 44318_2025_530_MOESM10_ESM.zip › Figure 4/4B/Tubulin-blot.tif]

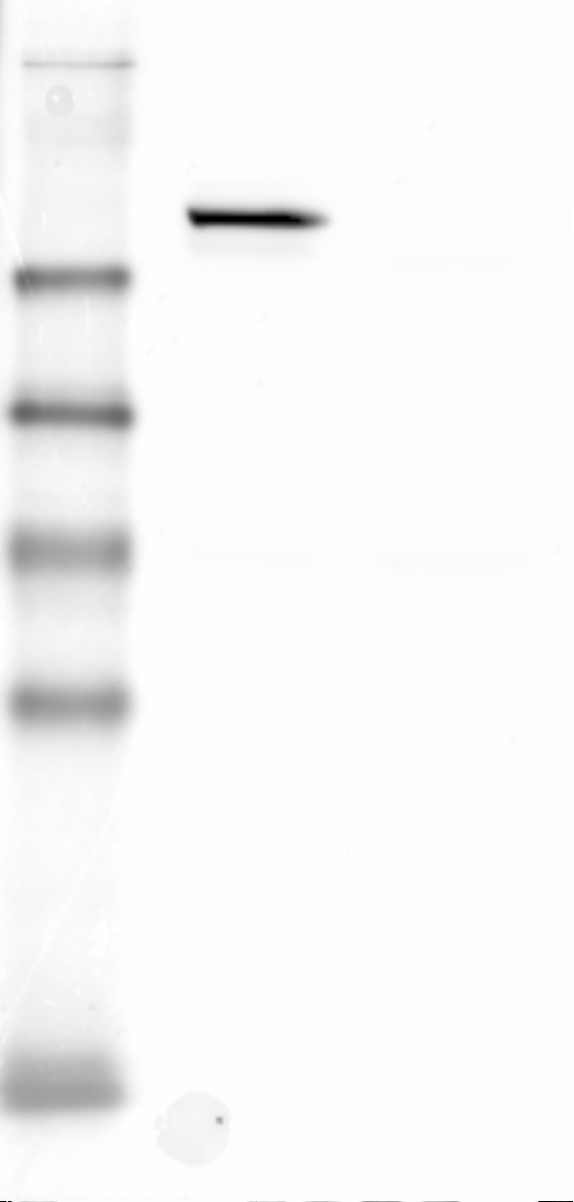

Supplement: Supplementary file 10 — Source data Fig. 4 [file 44318_2025_530_MOESM10_ESM.zip › Figure 4/4B/RABGAP1-KO-blot.tif]

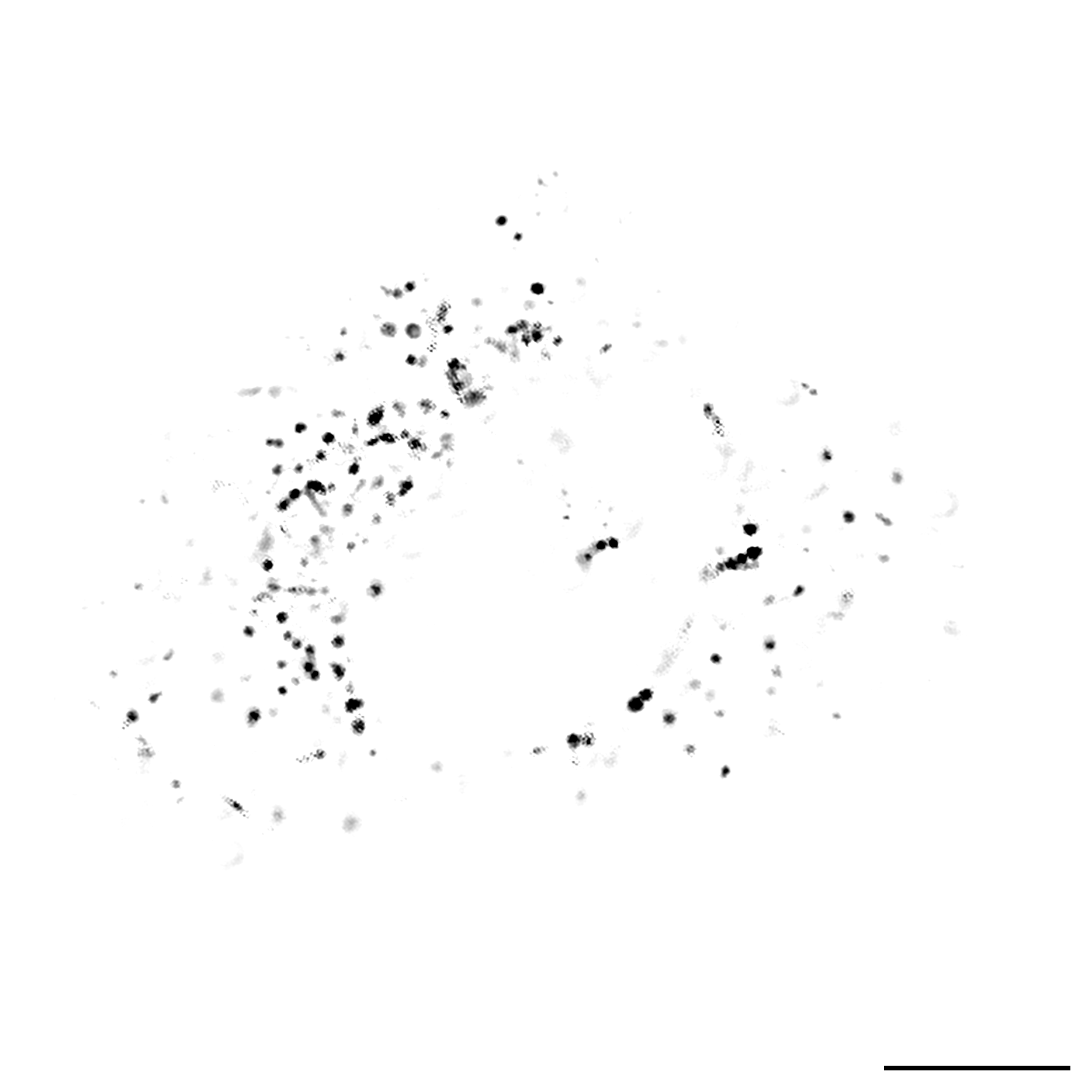

Supplement: Supplementary file 10 — Source data Fig. 4 [file 44318_2025_530_MOESM10_ESM.zip › Figure 4/4K/RABGAP1KO_HaloTag-APP.tif]

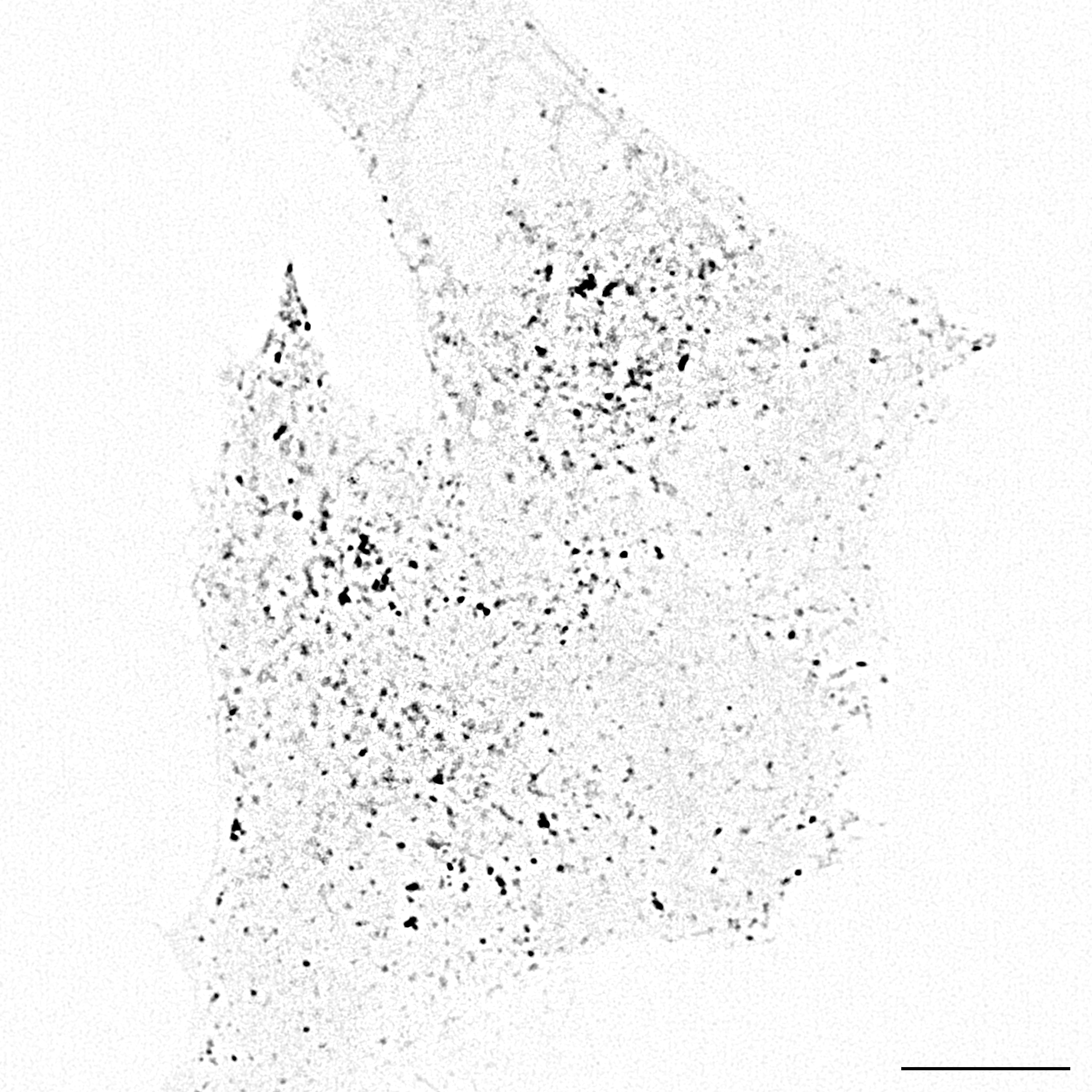

Supplement: Supplementary file 10 — Source data Fig. 4 [file 44318_2025_530_MOESM10_ESM.zip › Figure 4/4K/WT_HaloTag-APP.tif]

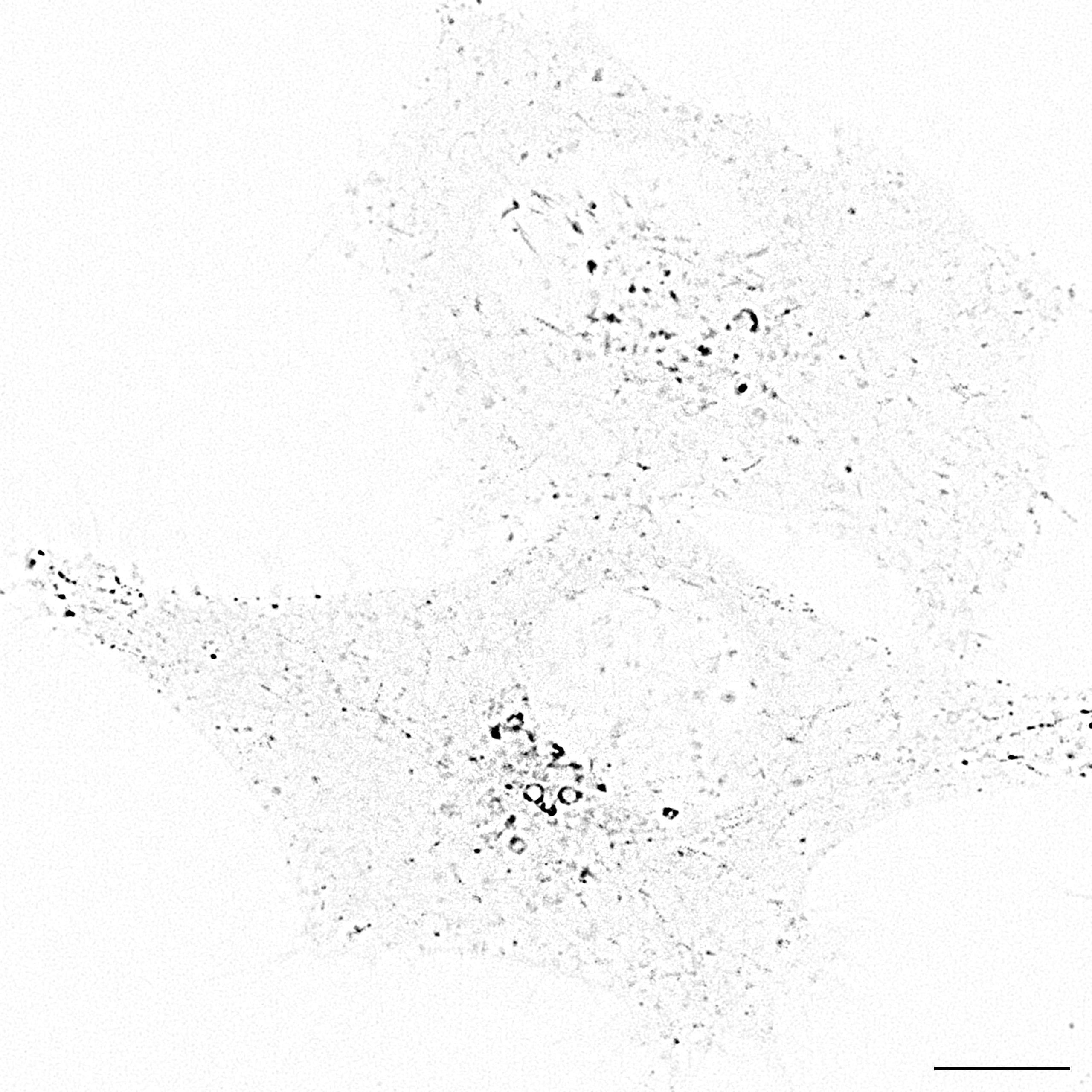

Supplement: Supplementary file 10 — Source data Fig. 4 [file 44318_2025_530_MOESM10_ESM.zip › Figure 4/4K/WT_RAB5A-mScarlet.tif]

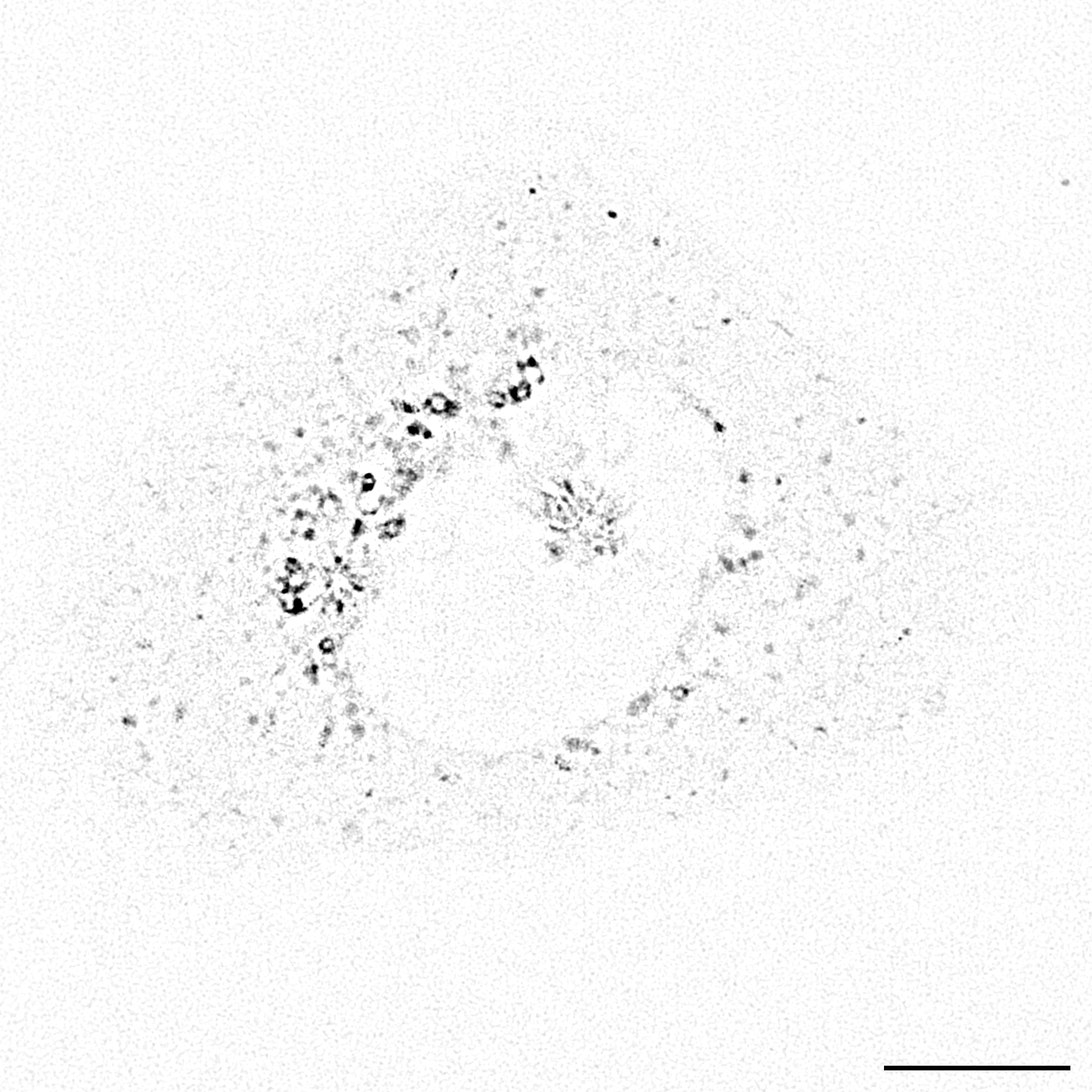

Supplement: Supplementary file 10 — Source data Fig. 4 [file 44318_2025_530_MOESM10_ESM.zip › Figure 4/4K/RABGAP1KO_RAB5A-mScarlet.tif]

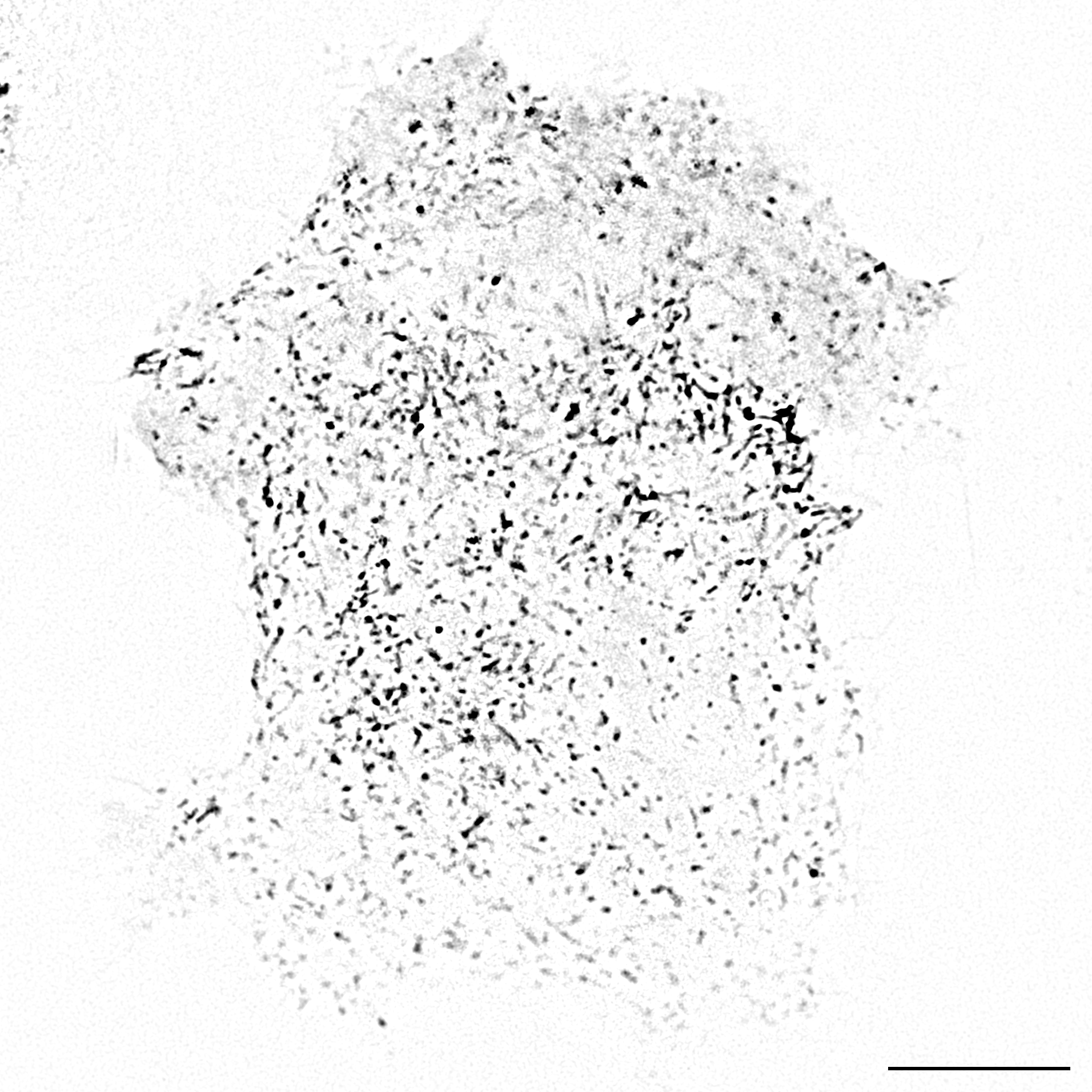

Supplement: Supplementary file 10 — Source data Fig. 4 [file 44318_2025_530_MOESM10_ESM.zip › Figure 4/4H/WT_RAB11A-mScarlet.tif]

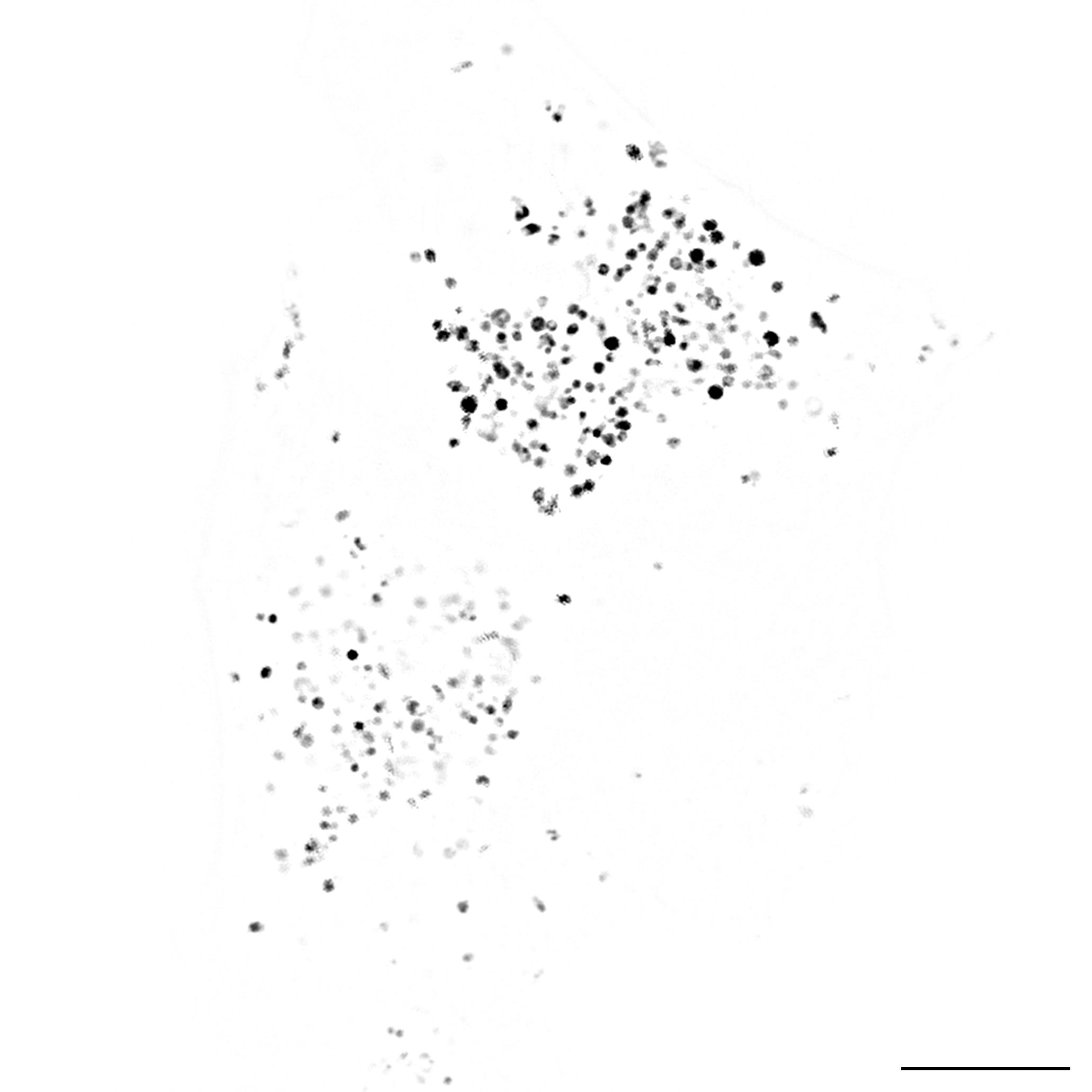

Supplement: Supplementary file 10 — Source data Fig. 4 [file 44318_2025_530_MOESM10_ESM.zip › Figure 4/4H/RABGAP1KO_HaloTag-APP.tif]

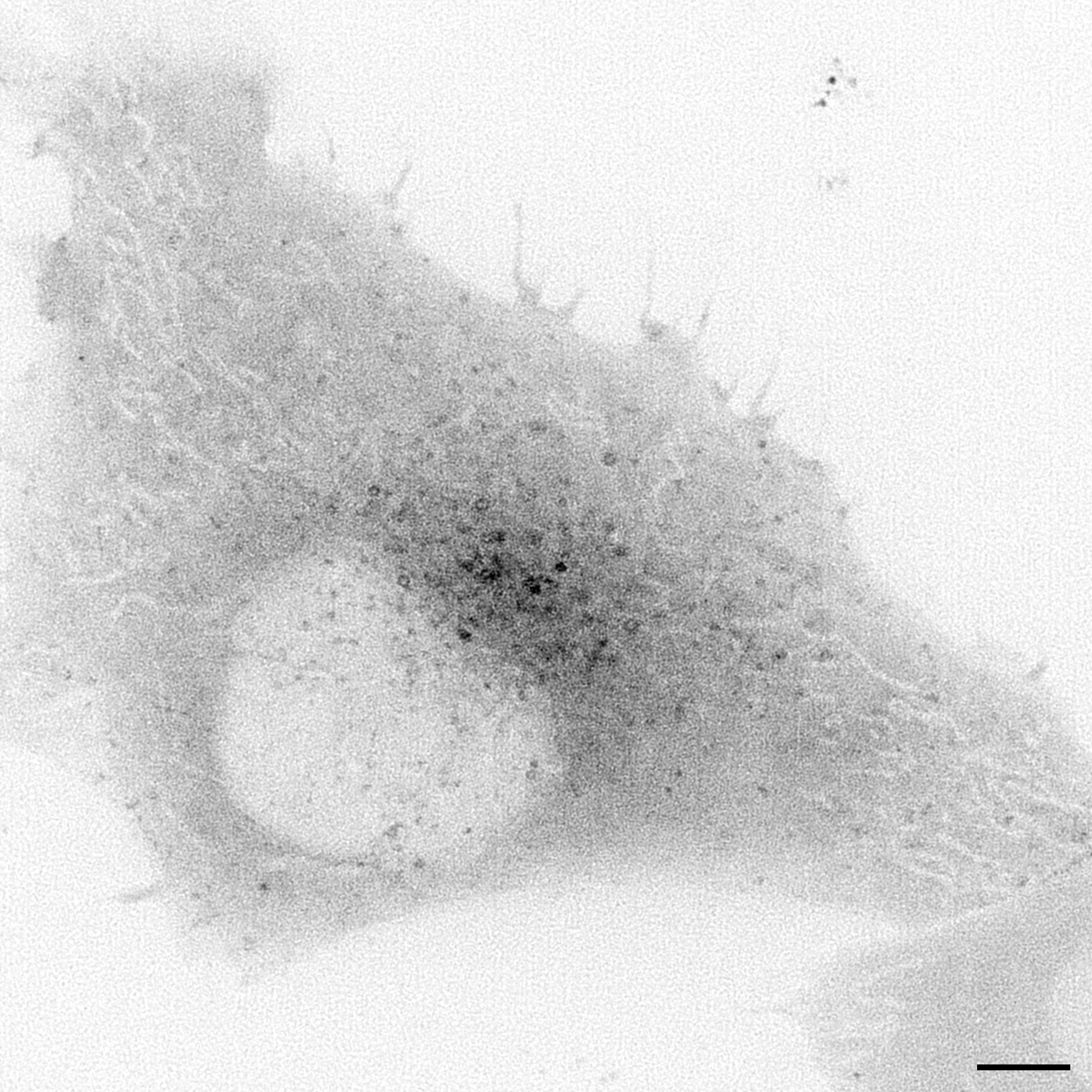

Supplement: Supplementary file 10 — Source data Fig. 4 [file 44318_2025_530_MOESM10_ESM.zip › Figure 4/4G/mCherry-RABGAP1.tif]

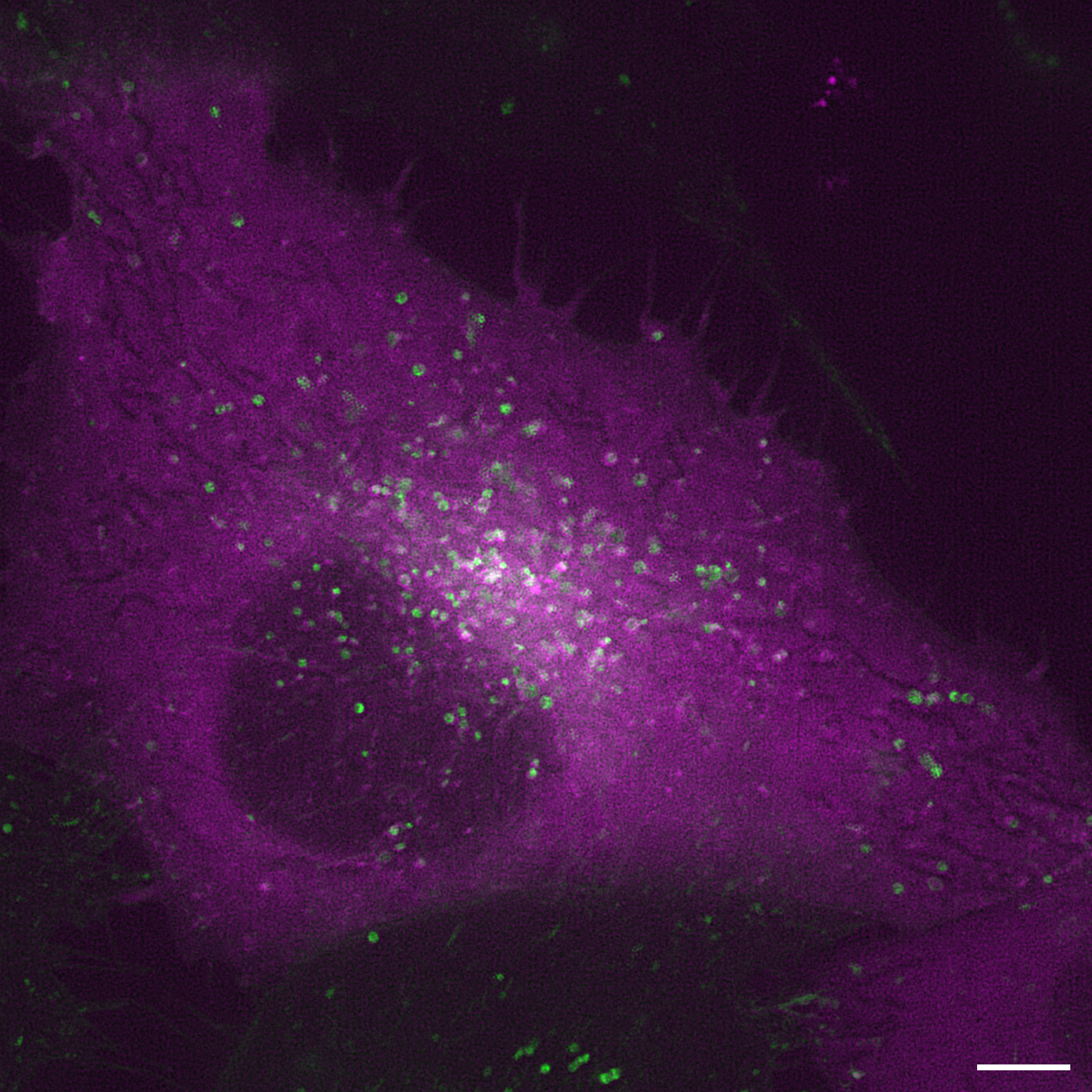

Supplement: Supplementary file 10 — Source data Fig. 4 [file 44318_2025_530_MOESM10_ESM.zip › Figure 4/4G/merged-image.tif]

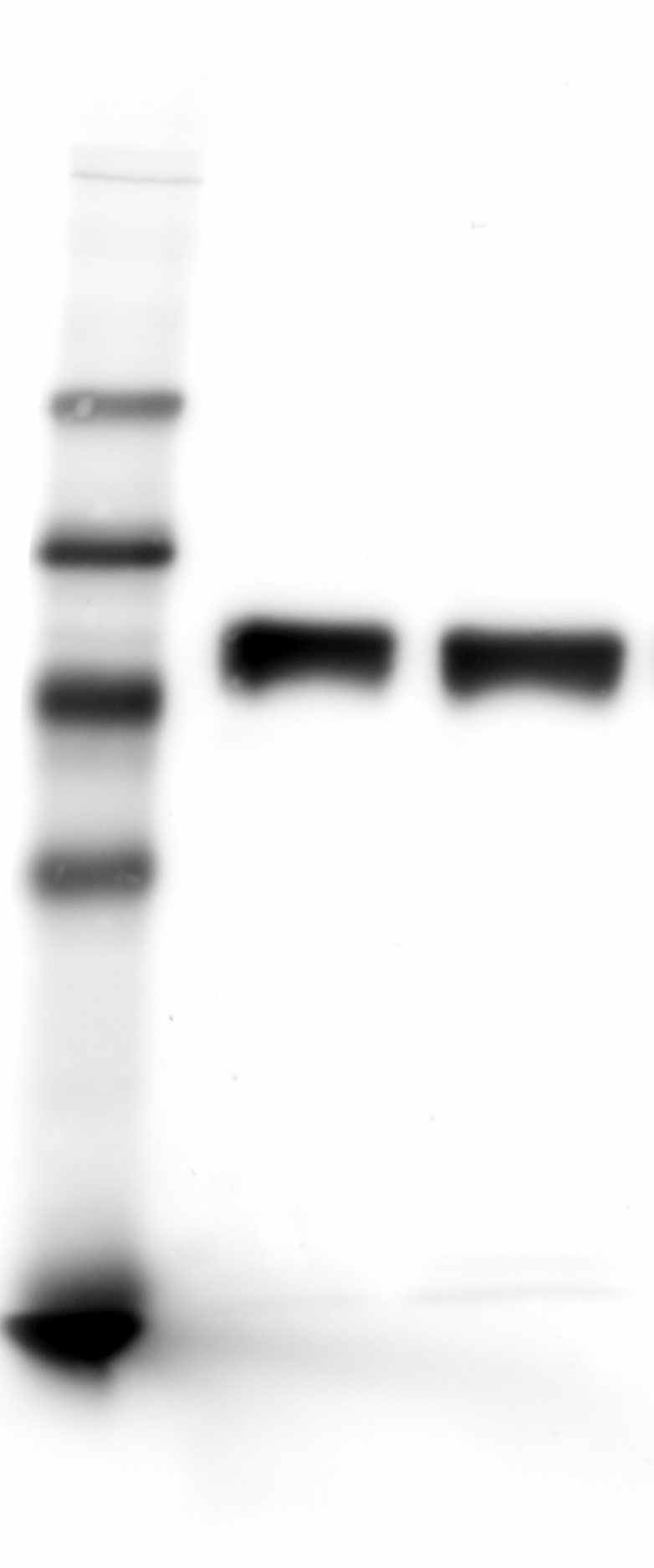

Supplement: Supplementary file 11 — Source data Fig. 5 [file 44318_2025_530_MOESM11_ESM.zip › Figure 5/5E/Tubulin.tif]

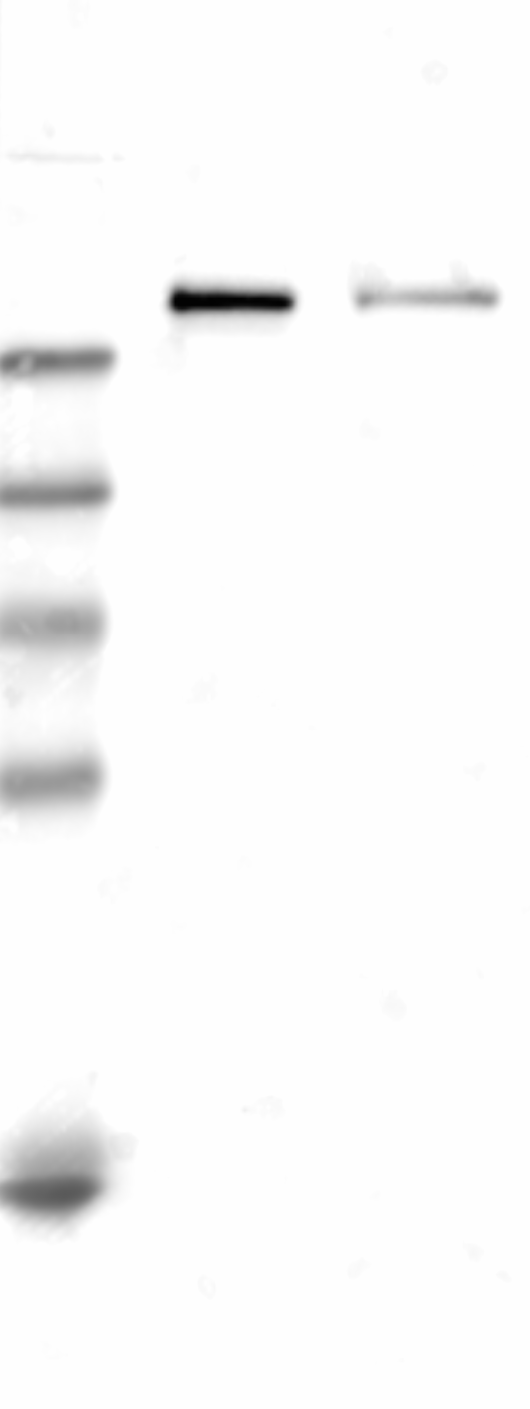

Supplement: Supplementary file 11 — Source data Fig. 5 [file 44318_2025_530_MOESM11_ESM.zip › Figure 5/5E/RABGAP1.tif]

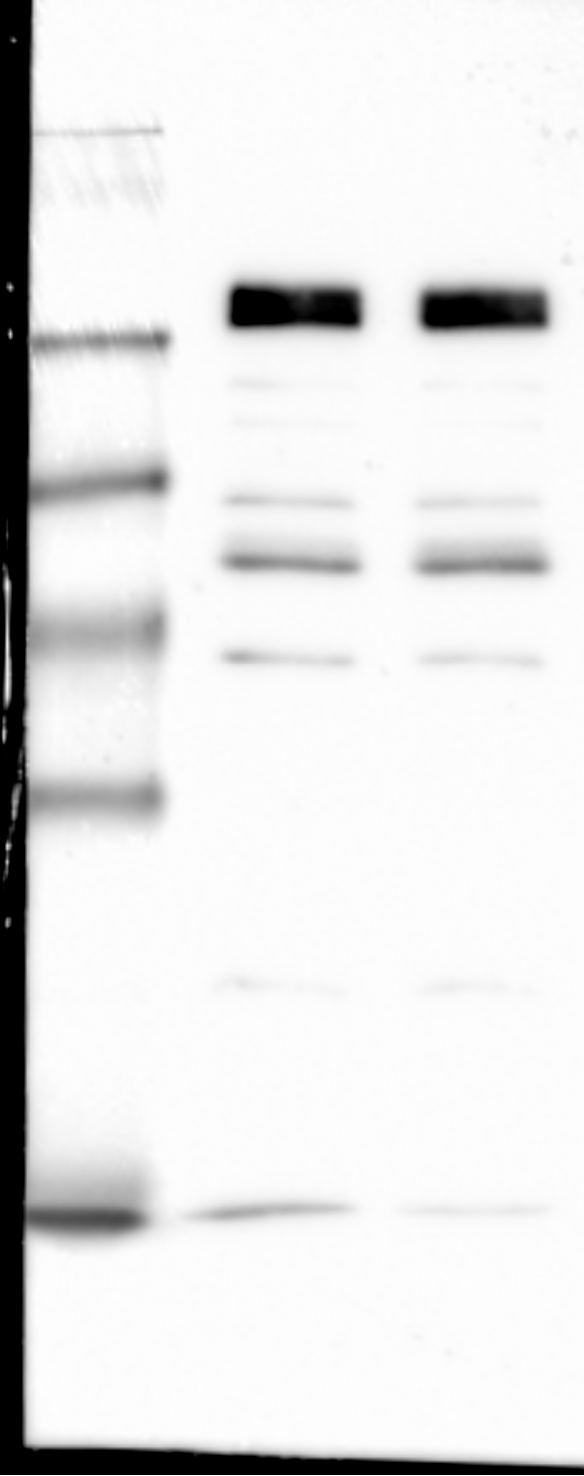

Supplement: Supplementary file 11 — Source data Fig. 5 [file 44318_2025_530_MOESM11_ESM.zip › Figure 5/5E/full-length-APP.tif]

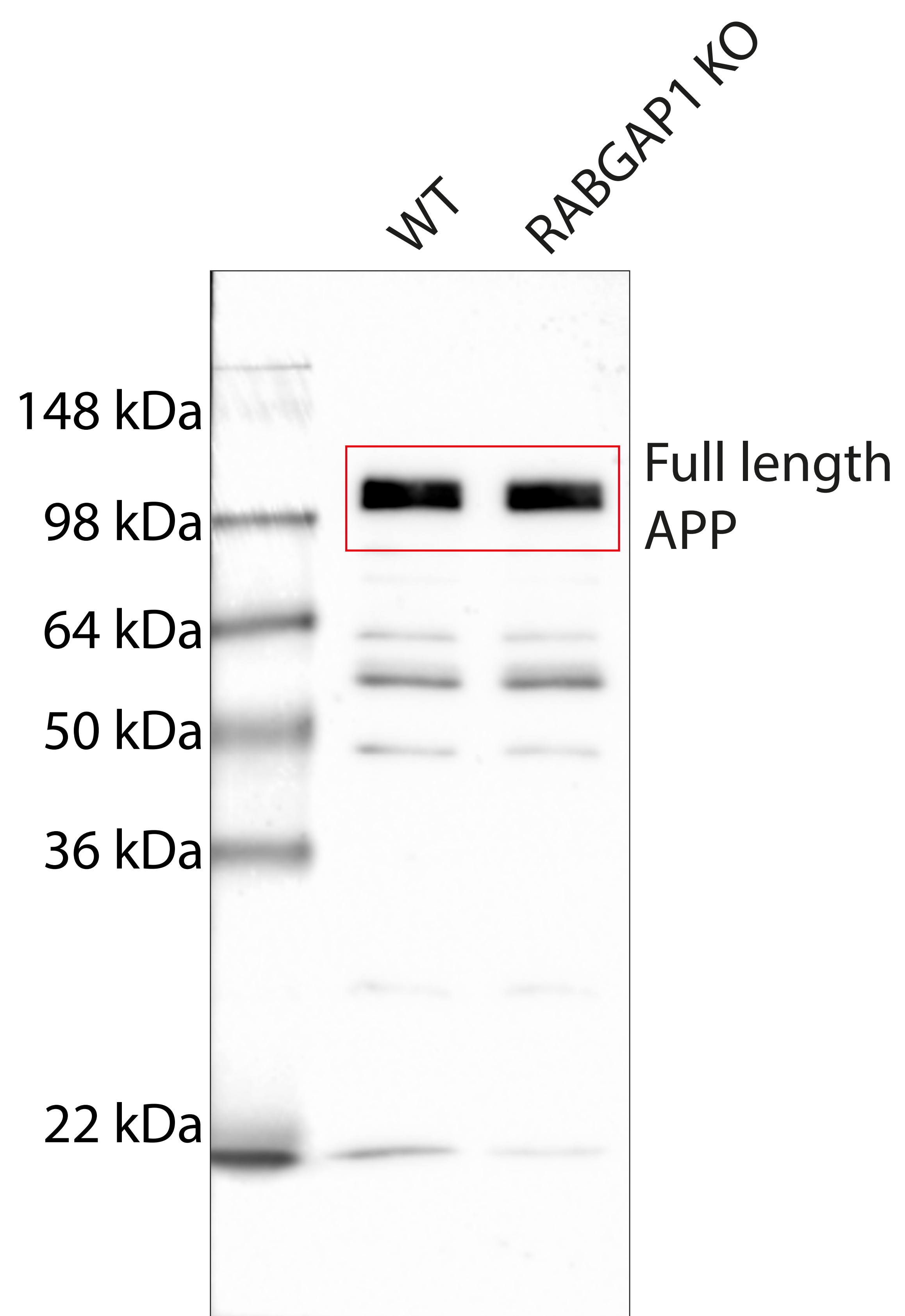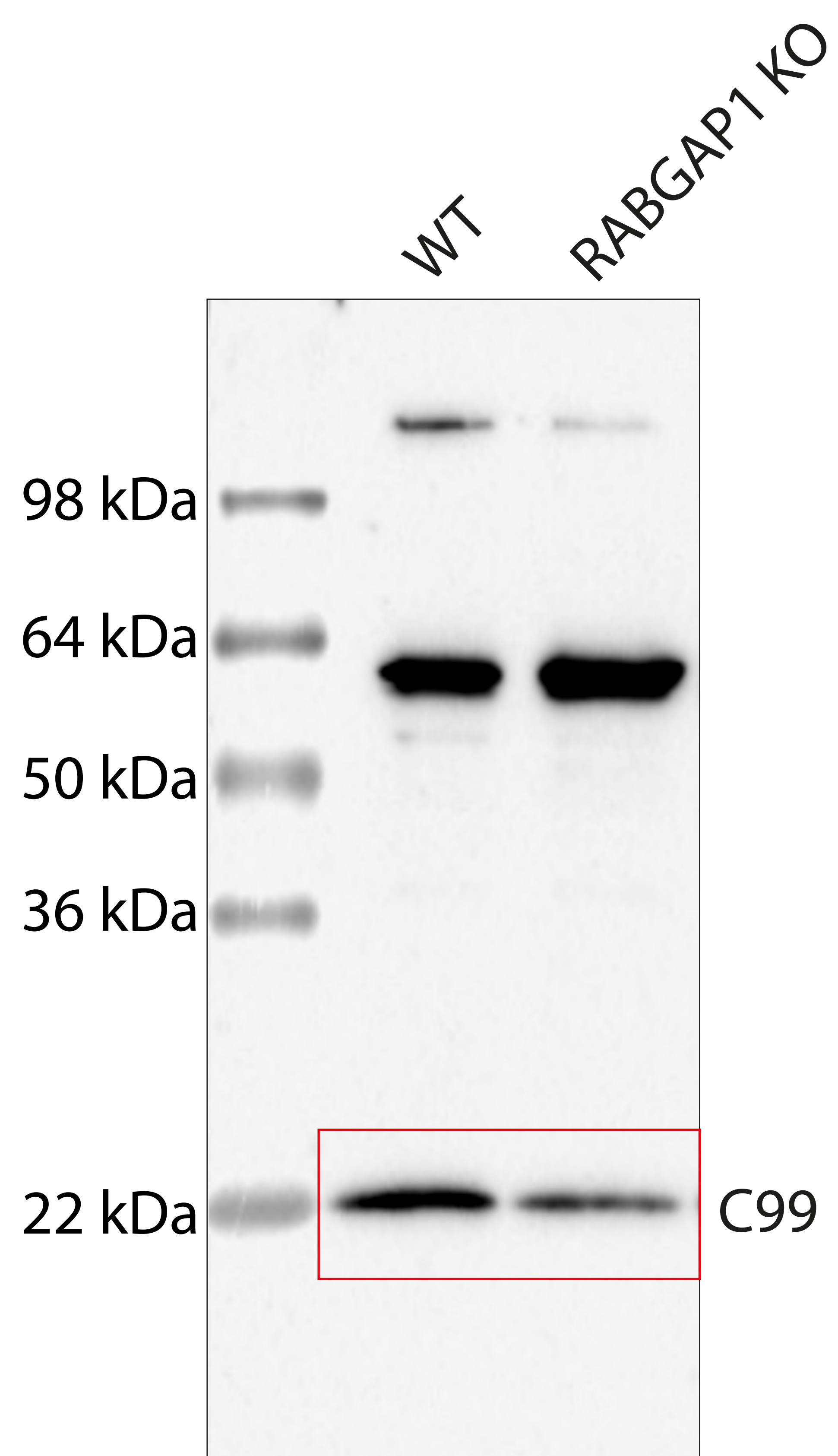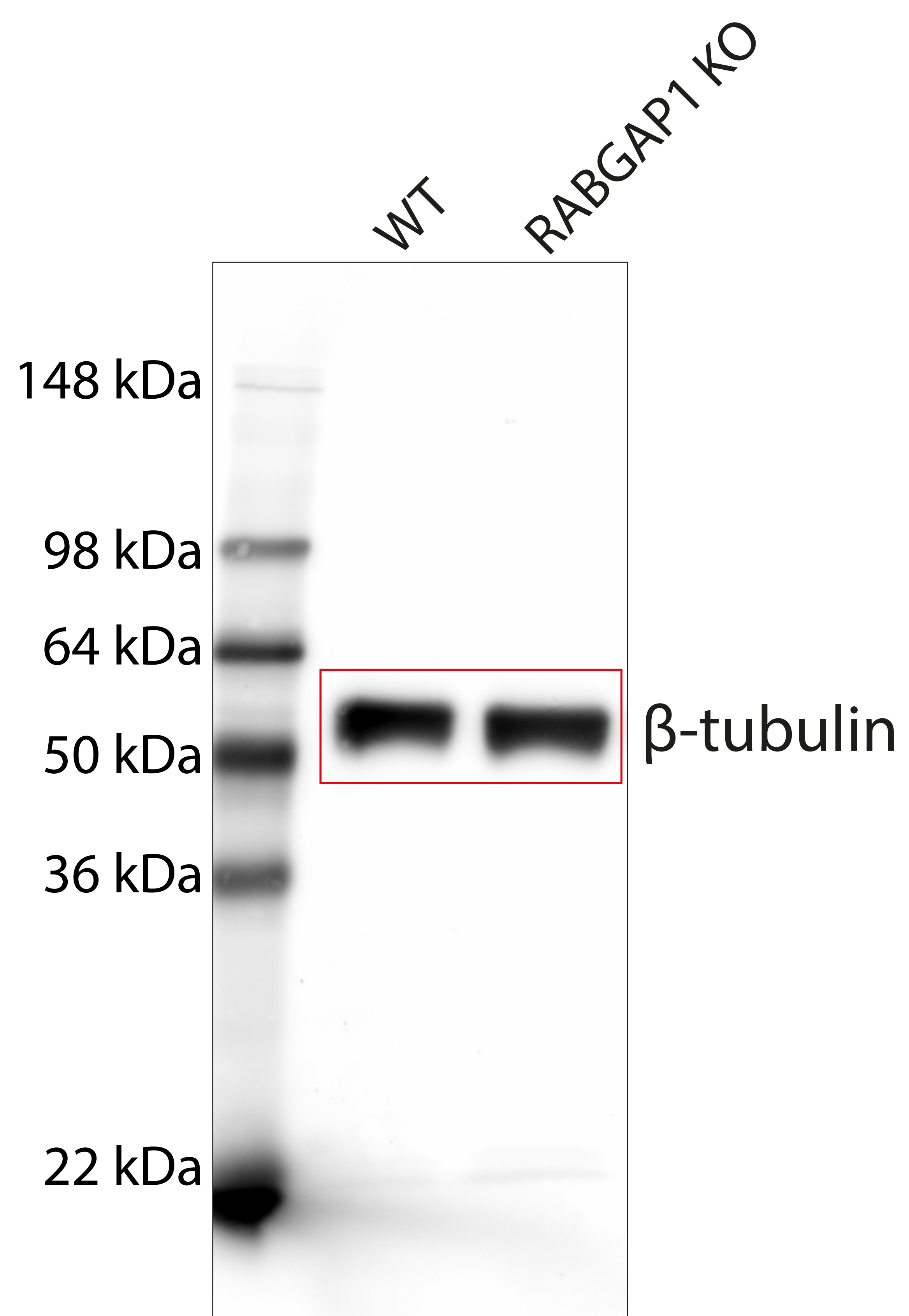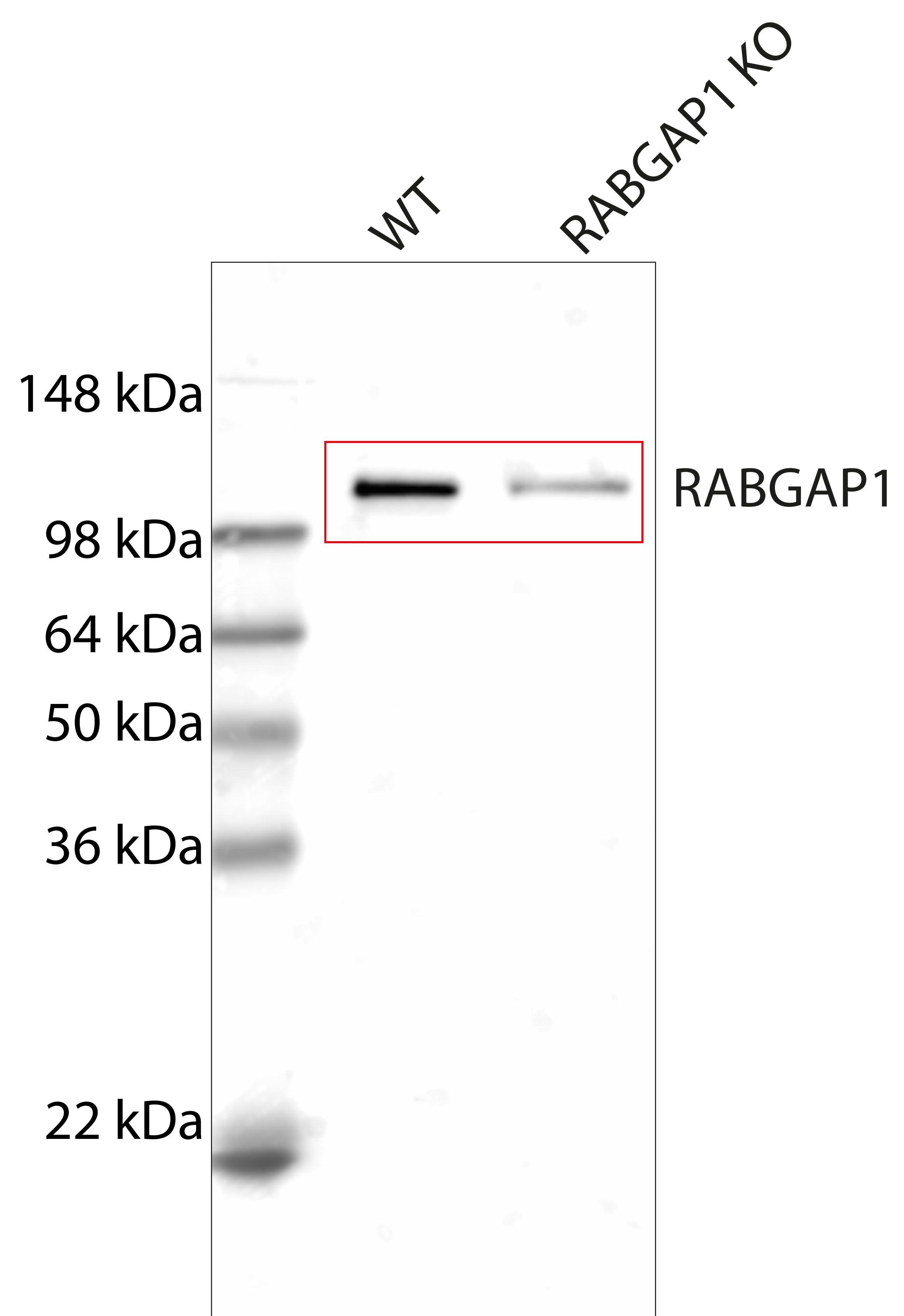

Supplement: Supplementary file 11 — Source data Fig. 5 [file 44318_2025_530_MOESM11_ESM.zip › Figure 5/5E/Figure 5E-blots.pdf]

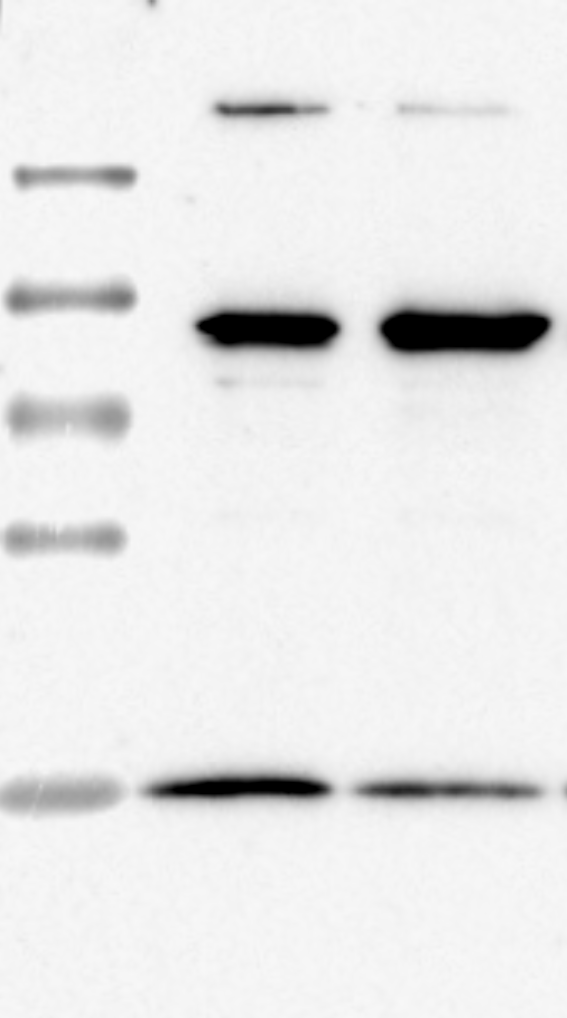

Supplement: Supplementary file 11 — Source data Fig. 5 [file 44318_2025_530_MOESM11_ESM.zip › Figure 5/5E/C99.tif]

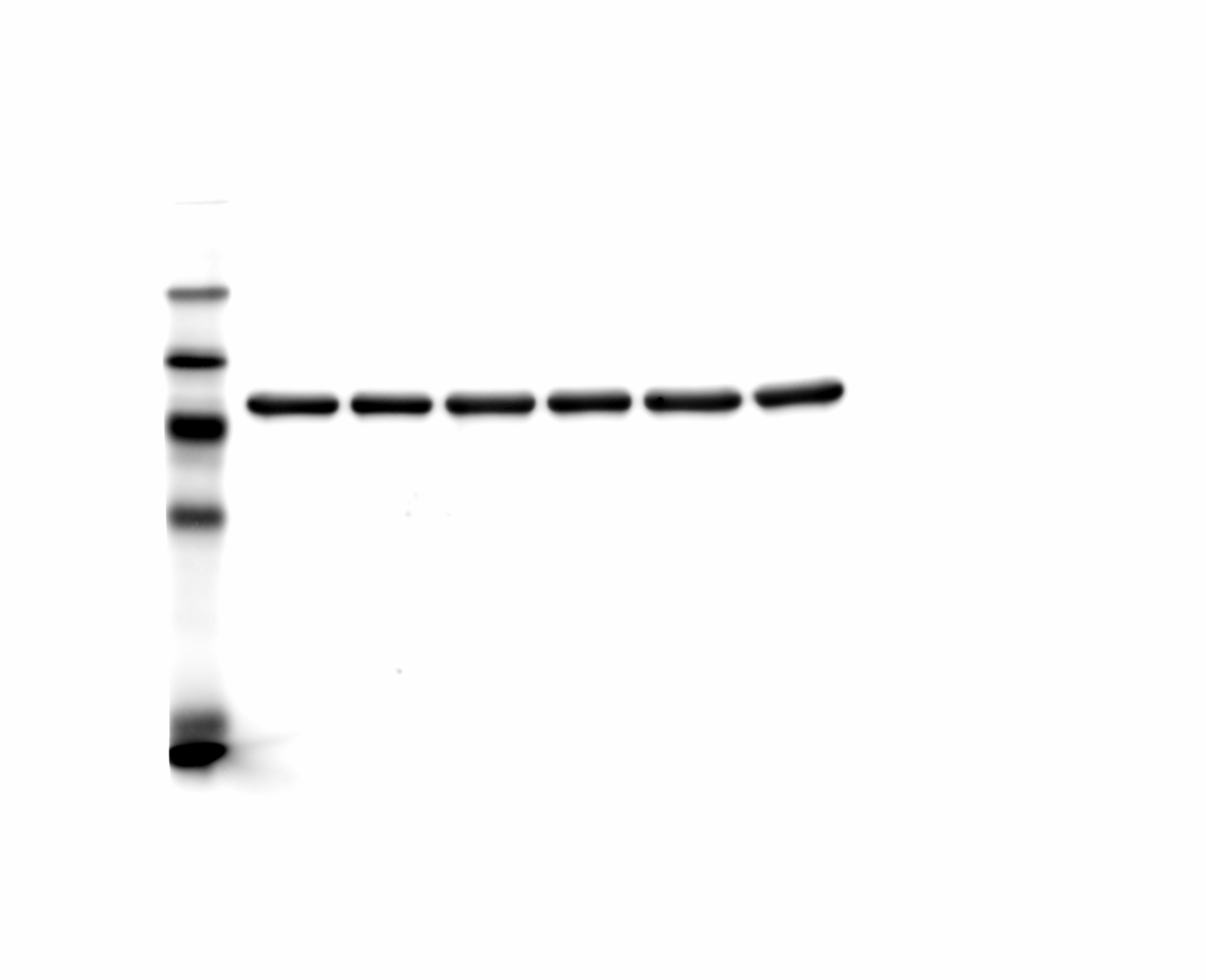

Supplement: Supplementary file 12 — Source data Fig. 6 [file 44318_2025_530_MOESM12_ESM.zip › Figure 6/6A/tubulin-recoveryblot.tif]

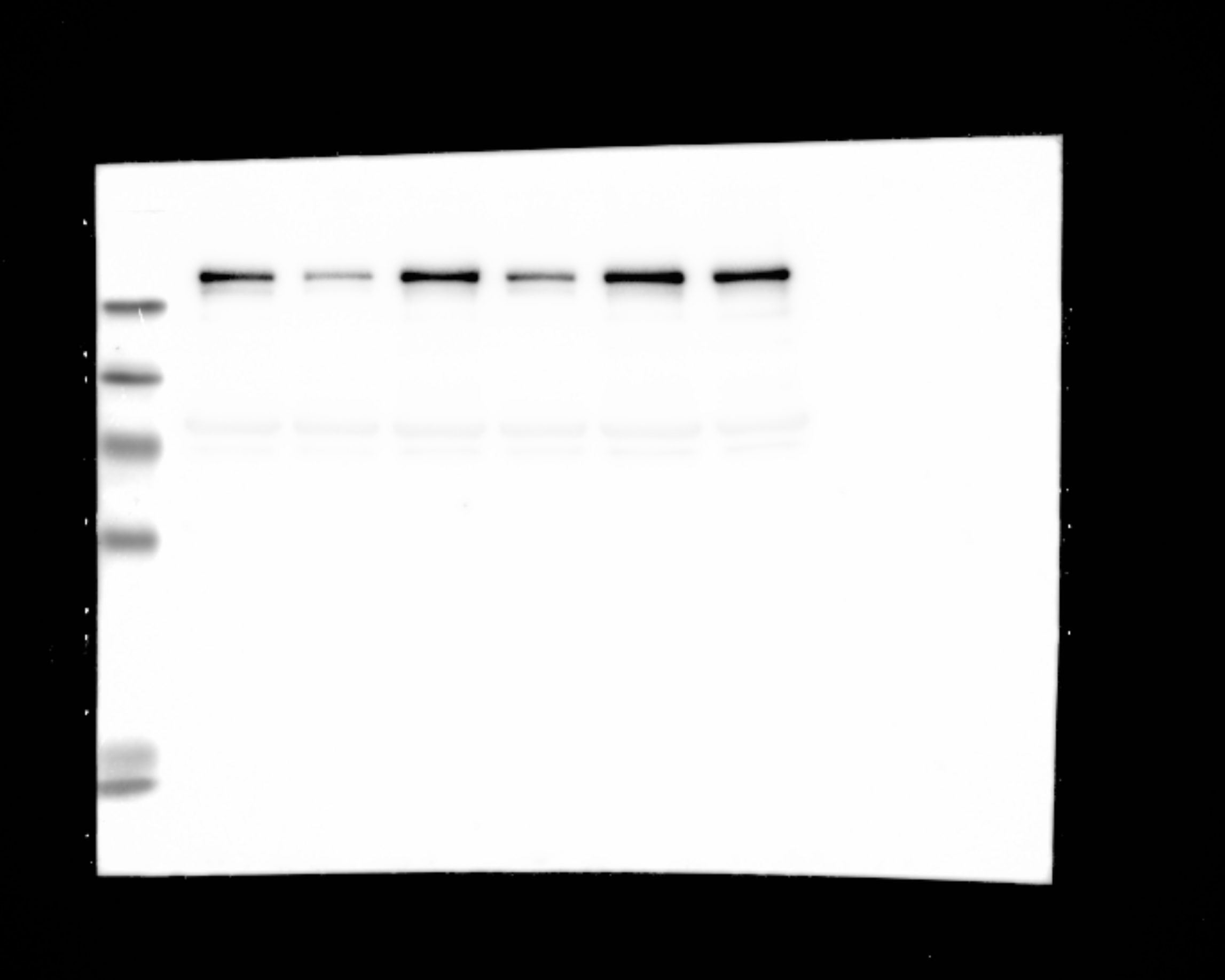

Supplement: Supplementary file 12 — Source data Fig. 6 [file 44318_2025_530_MOESM12_ESM.zip › Figure 6/6A/RABGAP1-recoveryblot.tif]

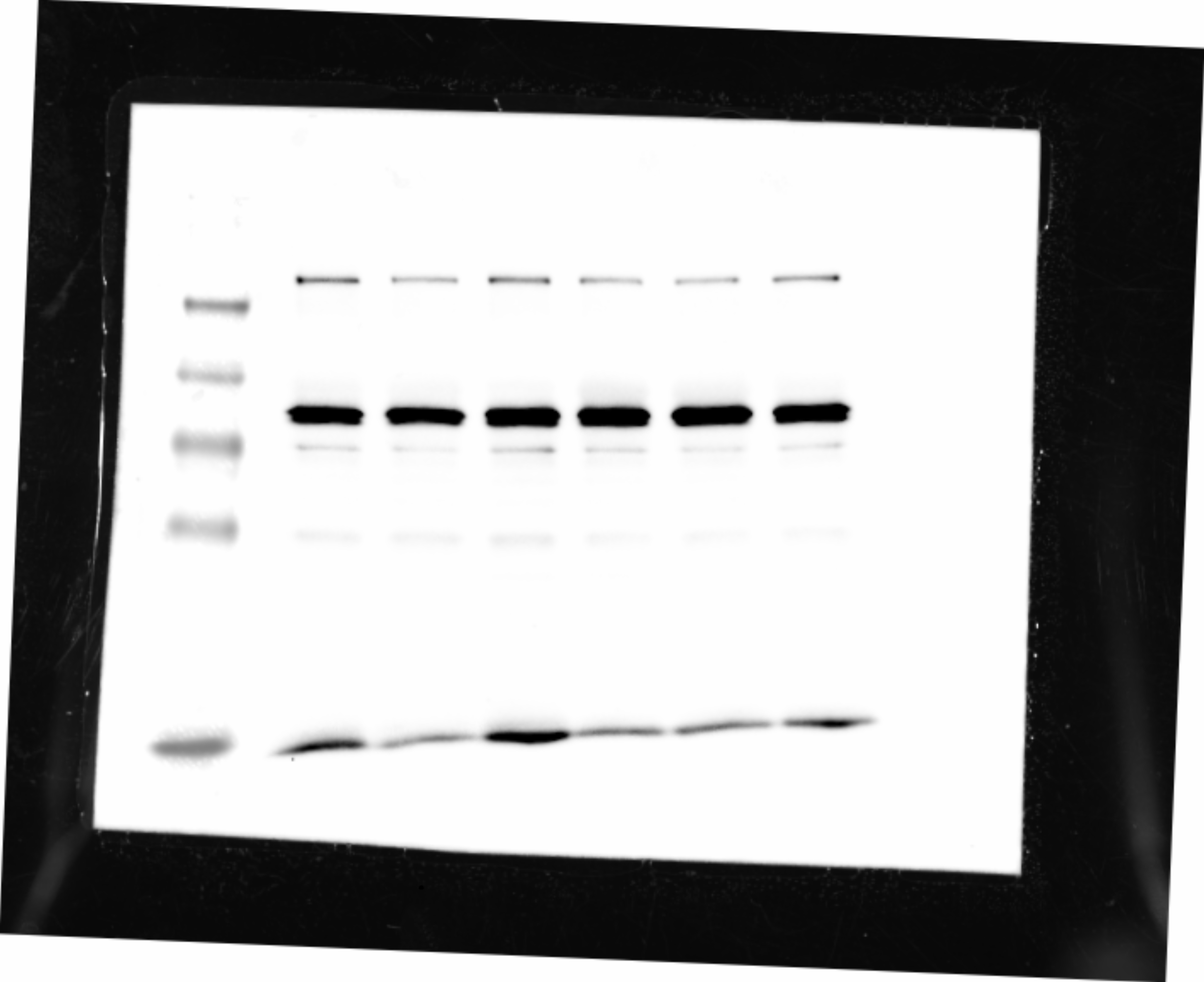

Supplement: Supplementary file 12 — Source data Fig. 6 [file 44318_2025_530_MOESM12_ESM.zip › Figure 6/6A/C99-recoveryblot.tif]

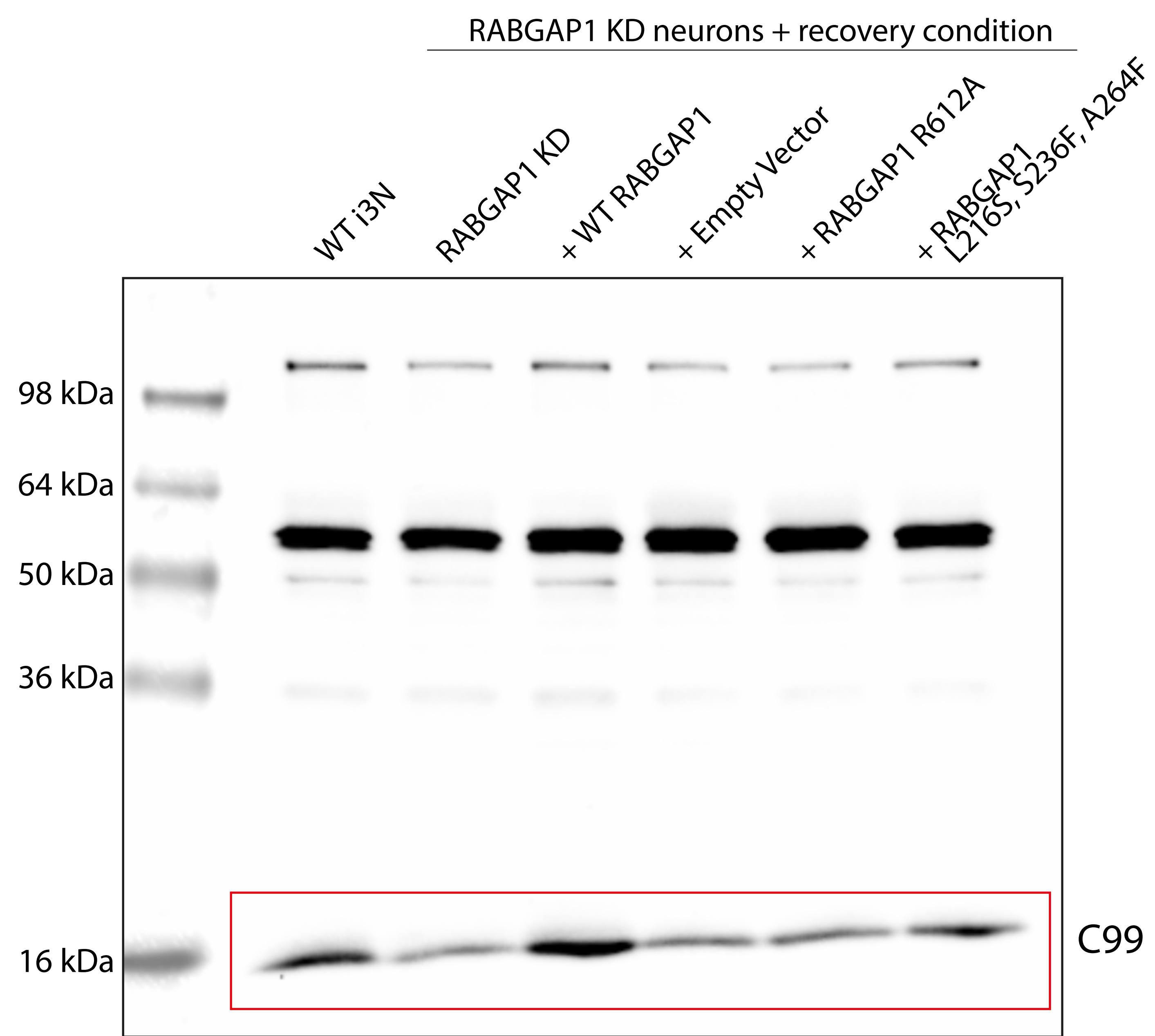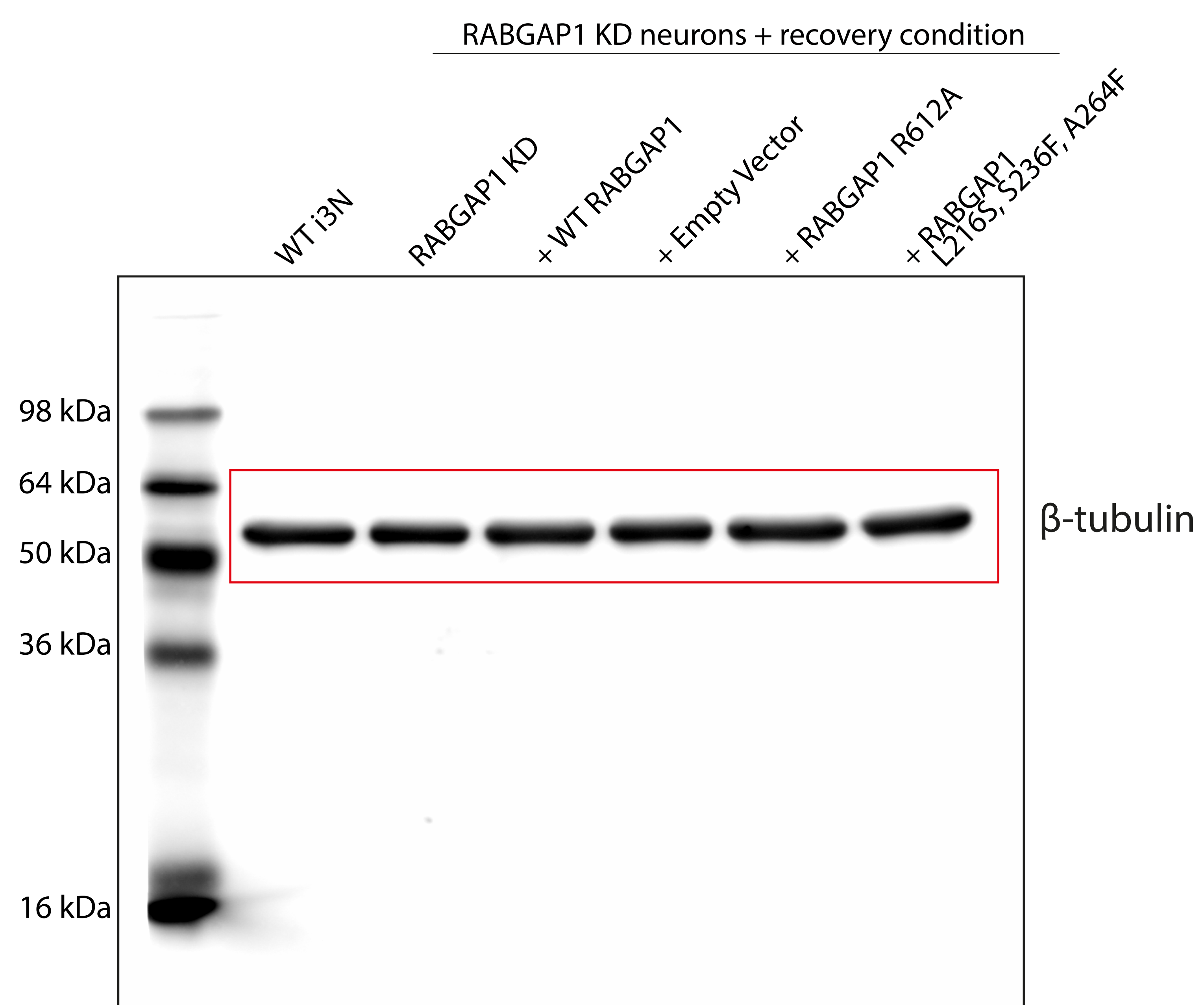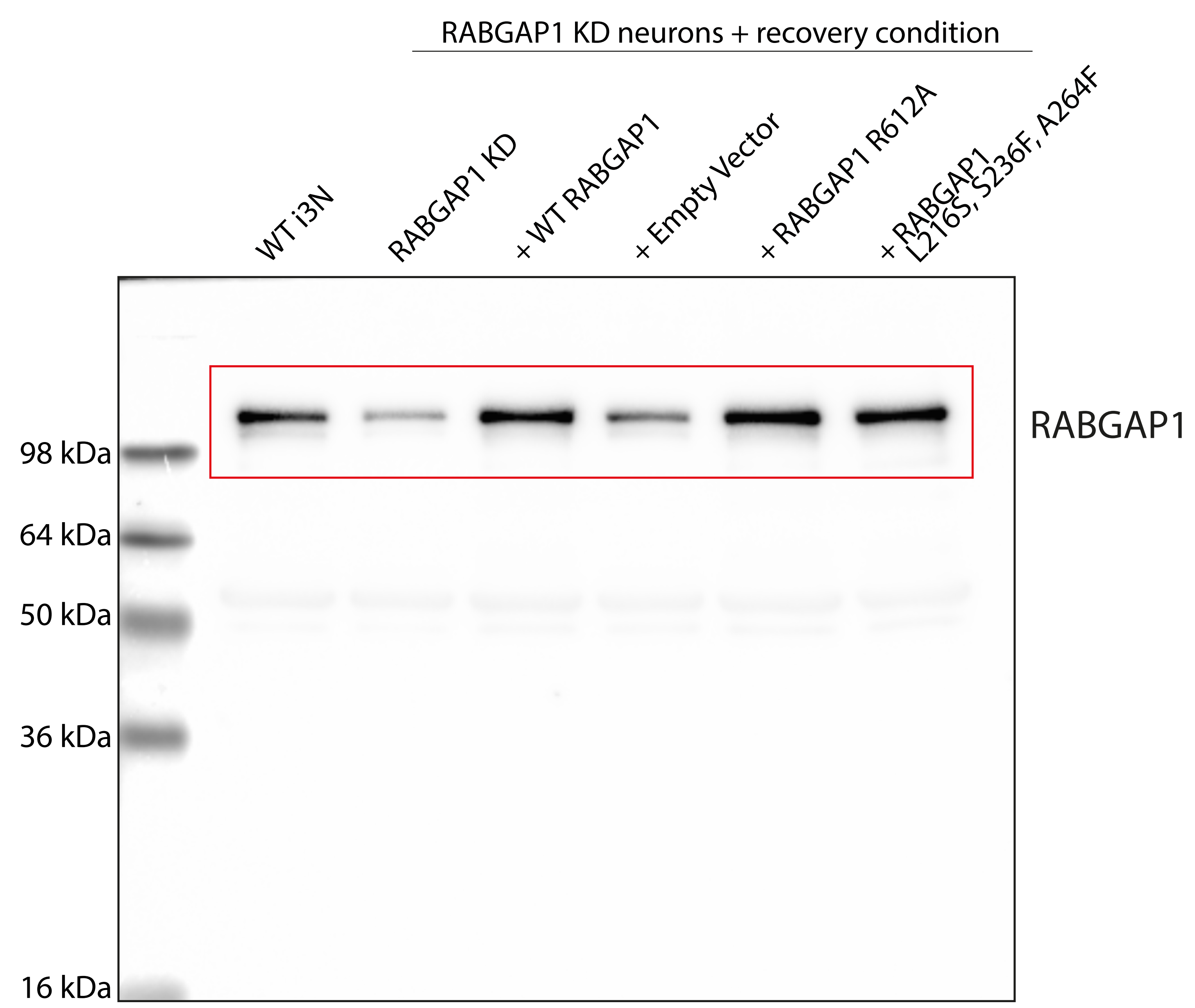

Supplement: Supplementary file 12 — Source data Fig. 6 [file 44318_2025_530_MOESM12_ESM.zip › Figure 6/6A/Figure 6A-blots.pdf]

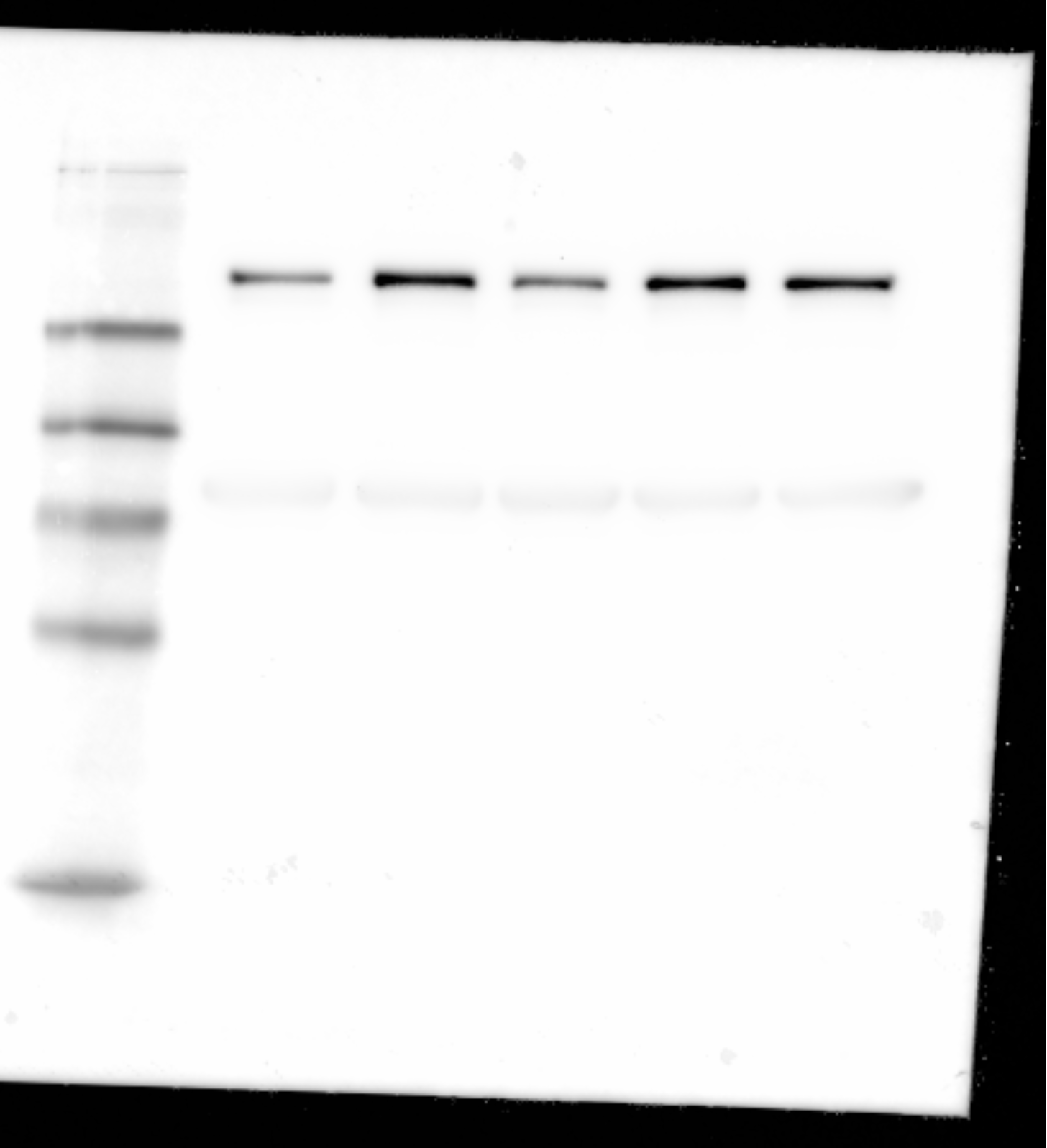

Supplement: Supplementary file 12 — Source data Fig. 6 [file 44318_2025_530_MOESM12_ESM.zip › Figure 6/6C/RABGAP1-overexpressionblot.tif]

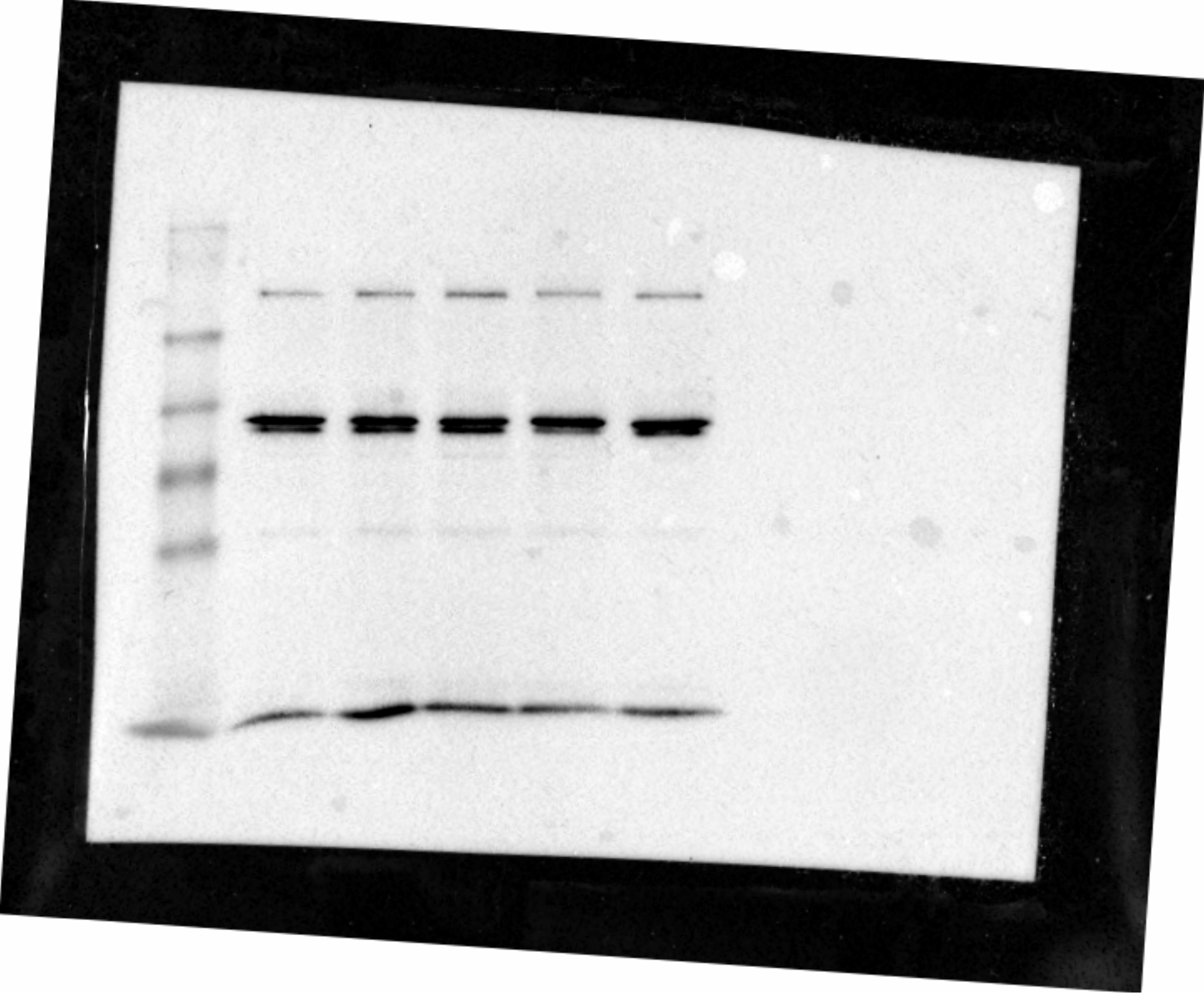

Supplement: Supplementary file 12 — Source data Fig. 6 [file 44318_2025_530_MOESM12_ESM.zip › Figure 6/6C/C99-overexpressionblot.tif]

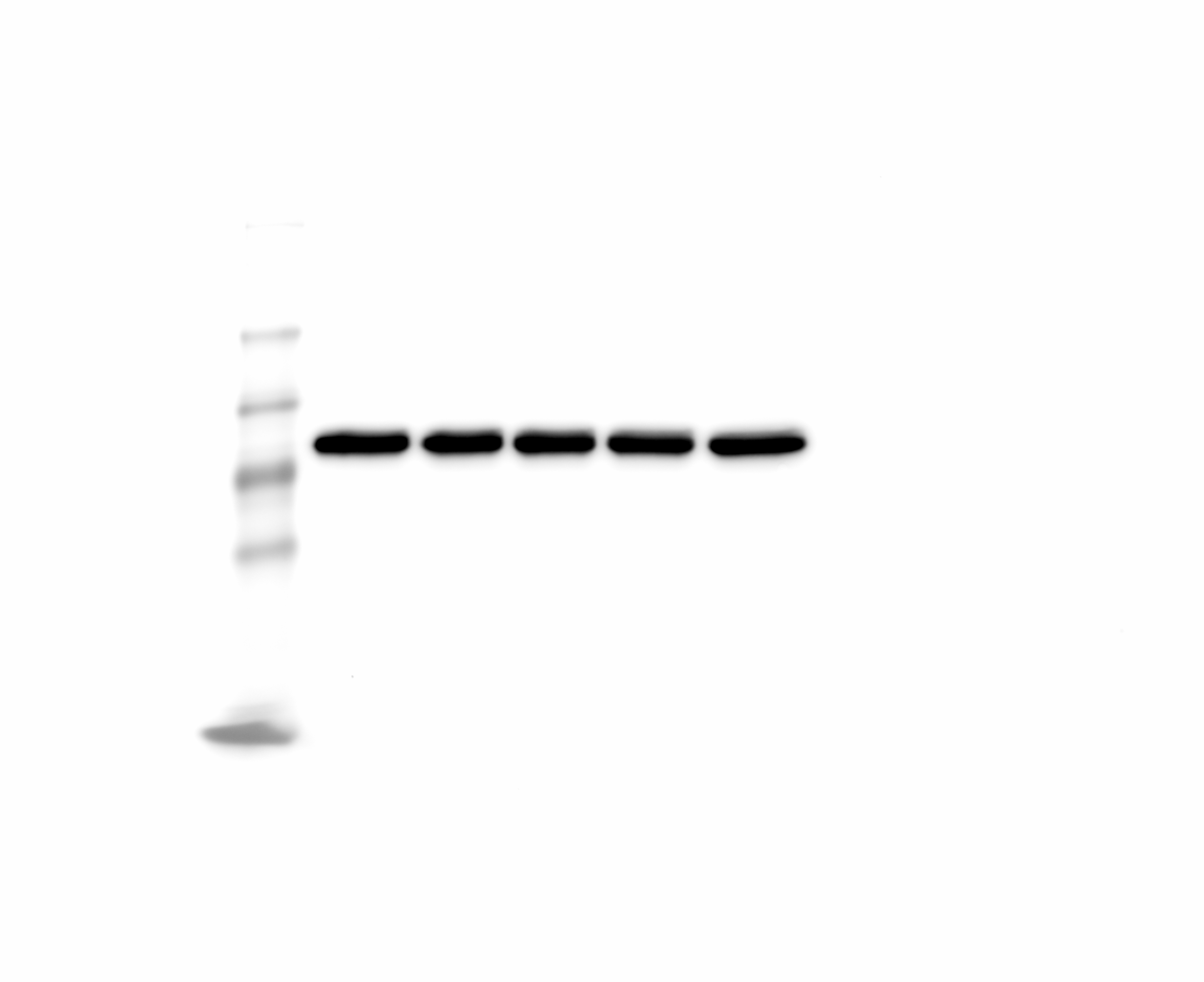

Supplement: Supplementary file 12 — Source data Fig. 6 [file 44318_2025_530_MOESM12_ESM.zip › Figure 6/6C/tubulin-overexpressionblot.tif]
