# Supplementary material for: RABGAP1 is a sensor that facilitates the sorting and processing of amyloid precursor protein
Source: EMBO J. 2025 Aug 26;44(19):5443–62. doi: 10.1038/s44318-025-00530-0 (PMC12489035; doi:10.1038/s44318-025-00530-0)
Supplement: Supplementary file 12 — Source data Fig. 6 [file 44318_2025_530_MOESM12_ESM.zip › Figure 6/6C/Figure 6B-blots.pdf]

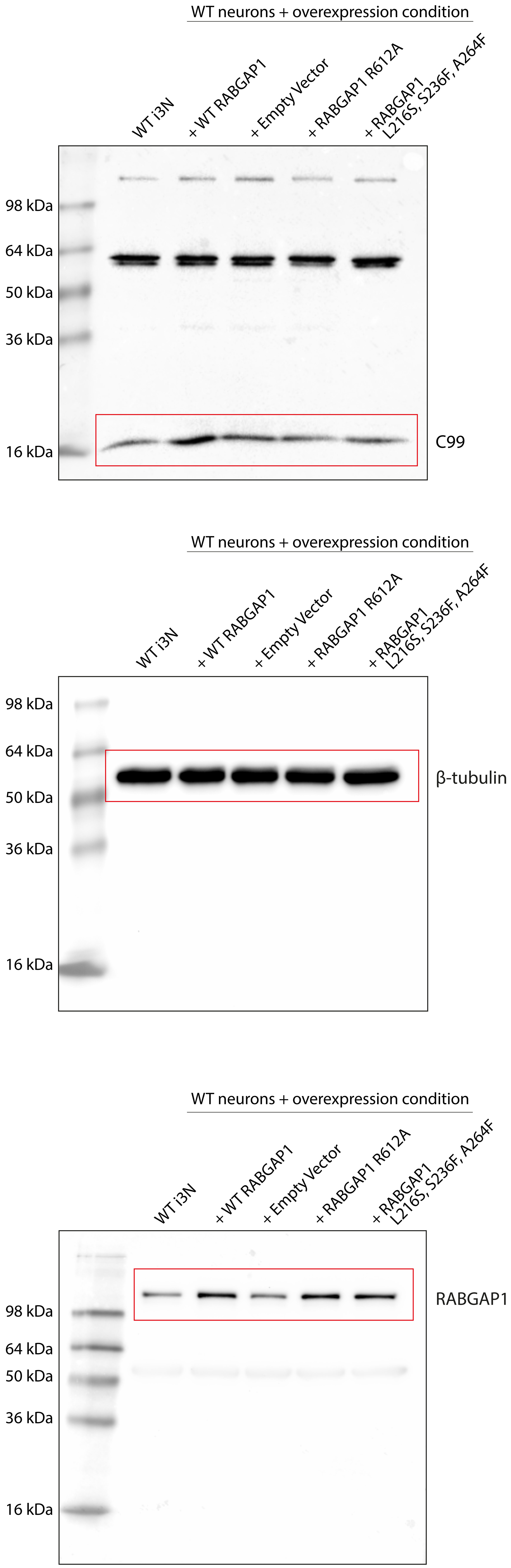
WT neurons + overexpression condition
WT i3N
+ WT RABGAP1
+ Empty Vector
+ RABGAP1 R612A
+ RABGAP1 L216S, S236F, A264F
98 kDa
64 kDa
50 kDa
36 kDa
16 kDa
C99
WT neurons + overexpression condition
WT i3N
+ WT RABGAP1
+ Empty Vector
+ RABGAP1 R612A
+ RABGAP1 L216S, S236F, A264F
98 kDa
64 kDa
50 kDa
36 kDa
16 kDa
β-tubulin
WT neurons + overexpression condition
WT i3N
+ WT RABGAP1
+ Empty Vector
+ RABGAP1 R612A
+ RABGAP1 L216S, S236F, A264F
98 kDa
64 kDa
50 kDa
36 kDa
16 kDa
RABGAP1
